# MEF2C and EBF1 Co-regulate B Cell-Specific Transcription

**DOI:** 10.1371/journal.pgen.1005845

**Published:** 2016-02-22

**Authors:** Nikki R. Kong, Matthew Davis, Li Chai, Astar Winoto, Robert Tjian

**Affiliations:** 1 Department of Molecular and Cell Biology, University of California, Berkeley, Berkeley, California, United States of America; 2 Department of Pathology, Brigham and Women’s Hospital, Boston, Massachusetts, United States of America; 3 Office of the President, Howard Hughes Medical Institute, Chevy Chase, Maryland, United States of America; Cincinnati Children's Hospital Medical Center, UNITED STATES

## Abstract

Hematopoietic stem cells are capable of self-renewal or differentiation along three main lineages: myeloid, erythroid, and lymphoid. One of the earliest lineage decisions for blood progenitor cells is whether to adopt the lymphoid or myeloid fate. Previous work had shown that myocyte enhancer factor 2C (MEF2C) is indispensable for the lymphoid fate decision, yet the specific mechanism of action remained unclear. Here, we have identified early B cell factor-1 (EBF1) as a co-regulator of gene expression with MEF2C. A genome-wide survey of MEF2C and EBF1 binding sites identified a subset of B cell-specific genes that they target. We also determined that the p38 MAPK pathway activates MEF2C to drive B cell differentiation. *Mef2c* knockout mice showed reduced B lymphoid-specific gene expression as well as increased myeloid gene expression, consistent with MEF2C’s role as a lineage fate regulator. This is further supported by interaction between MEF2C and the histone deacetylase, HDAC7, revealing a likely mechanism to repress the myeloid transcription program. This study thus elucidates both activation and repression mechanisms, identifies regulatory partners, and downstream targets by which MEF2C regulates lymphoid-specific differentiation.

## Author Summary

B cells comprise important defense systems against infections in animals. Generating B cells requires the interplay of signals received by a blood stem cell and the ability of this cell to turn on or off gene expression, the latter of which is regulated largely by transcription factors. Despite the characterization of many transcription factors and their functions in B cell differentiation, there still remains an incomplete understanding of how these molecules work together and the hierarchy involved in cell lineage determination. Mis-regulation by transcription factors can lead to many blood disorders such as leukemias and lymphomas, making the discovery of the missing links in transcription regulation important. This study places the transcription factor MEF2C at the node of the complex gene expression network that determines the B cell fate. We identified many new transcriptional targets of MEF2C, elucidated the signal to activate its function, as well as offered insights on how MEF2C can balance its dual role to both turn on and off gene expression. In summary, this study contributed to understanding the important molecular network underlying the generation of B cells.

## Introduction

Hematopoiesis is the process that generates all blood cell types throughout the lifetime of an animal. Maintenance of homeostasis in blood cell differentiation is crucial for the organism to fight against infections while also transporting oxygen throughout the body. The rapid turnover of blood cells requires the rare hematopoietic stem cells (HSCs) to self-renew in their bone marrow niche, and differentiate when induced by a milieu of cytokines and signaling pathways [1]. HSCs differentiate along three main pathways: myeloid, lymphoid, and erythroid [2], any of which requires an intricate coordination of signal relay and transcriptional regulation. One of the earliest lineage choices for differentiating HSCs is to adopt the lymphoid or myeloid fate. Several transcription factors involved in this choice have been identified. For example, CCAAT/enhancer binding protein alpha (C/EBPα) (GenBank EDL03027.1) acts as the “master” myeloid regulator [3] [4], and E2A proteins—E12 (UniProt E9PWE2) and E47 (UniProt E9PVV2) isoforms—function as key transcription factors for the lymphoid fates [5,6]. Although they do not display B cell-specific expression, E2A proteins are known to activate important B lineage transcription factors such as early B cell factor-1 (EBF1) [7,8]. To more fully understand the gene regulatory network driving B cell differentiation, it becomes important to identify additional factors that activate the transcription program for B cell differentiation, especially those factors that are activated prior to the lymphoid fate commitment. Myocyte enhancer factor 2C (MEF2C) was a likely candidate to drive this process.

MEF2C is a member of MADS (MCM1, Agamous, Deficiens, Serum response factor)-box DNA binding domain-containing family of transcription factors [9] originally identified in skeletal and cardiac muscle development [10]. MEF2C is the only isoform in the MEF2 family whose expression in blood cells is restricted to B lymphocytes [11]. Conditional knockouts at different developmental stages have been generated from mice with a floxed *Mef2c* exon 2, which encodes the MADS DNA-binding and dimerization domains [12]. *Mef2c*^fl/fl^ *Vav1*-Cre mice, where recombination occurs after fetal hematopoiesis, showed slightly decreased numbers of peripheral blood B cells in younger mice but dramatically decreased early B cell populations in the bone marrow of older mice [13]. Deletion of MEF2C during early B cell development with *Mb1*-Cre also showed a delay in B cell development with down-regulation of some key B cell genes [14]. *Mef2c*^fl/fl^ *Mx1*-Cre mice (inducible deletion in all hematopoietic lineages) showed decreased numbers of common lymphoid progenitors (CLPs) and very low numbers of B cells but increased myeloid cell numbers [15]. Together, these studies present evidence that MEF2C is a lineage-restricting transcription factor that directs multi-potent hematopoietic progenitors to differentiate into the B cell lineage. MEF2C has also been reported to be required for B cell function as shown by two different studies using B cell-specific *Cd19*-Cre mice. However, these studies have suggested seemingly conflicting mechanisms for MEF2C activation: either via p38 MAPK-dependent phosphorylation [16] [17] or through calcium-dependent calcineurin-calmodulin pathways, which release MEF2C from binding by class II HDACs [18] [19]. Thus, despite a long-standing interest in this important regulatory factor, how MEF2C transcriptionally directs lymphoid specification while repressing the myeloid fate, as well as the identities of its downstream targets and the modes of its activation in B cells remained largely unknown or unclear.

Here, we have identified a transcriptional co-regulatory partner of MEF2C, EBF1. They co-immunoprecipitate (IP) and the cooperation is important to turn on several B cell-specific target genes that we have identified by genome-wide chromatin IP (ChIP) experiments. In addition, our studies suggest that the p38 MAPK pathway is responsible for activating MEF2C in the lymphoid versus myeloid fate decision. Our findings thus elucidate a novel co-regulator, downstream target genes, and the mechanisms of activation by which MEF2C drives lymphoid-specific hematopoietic differentiation.

## Results

### MEF2C specifically co-immunoprecipitates with EBF1

**Fig 1:**
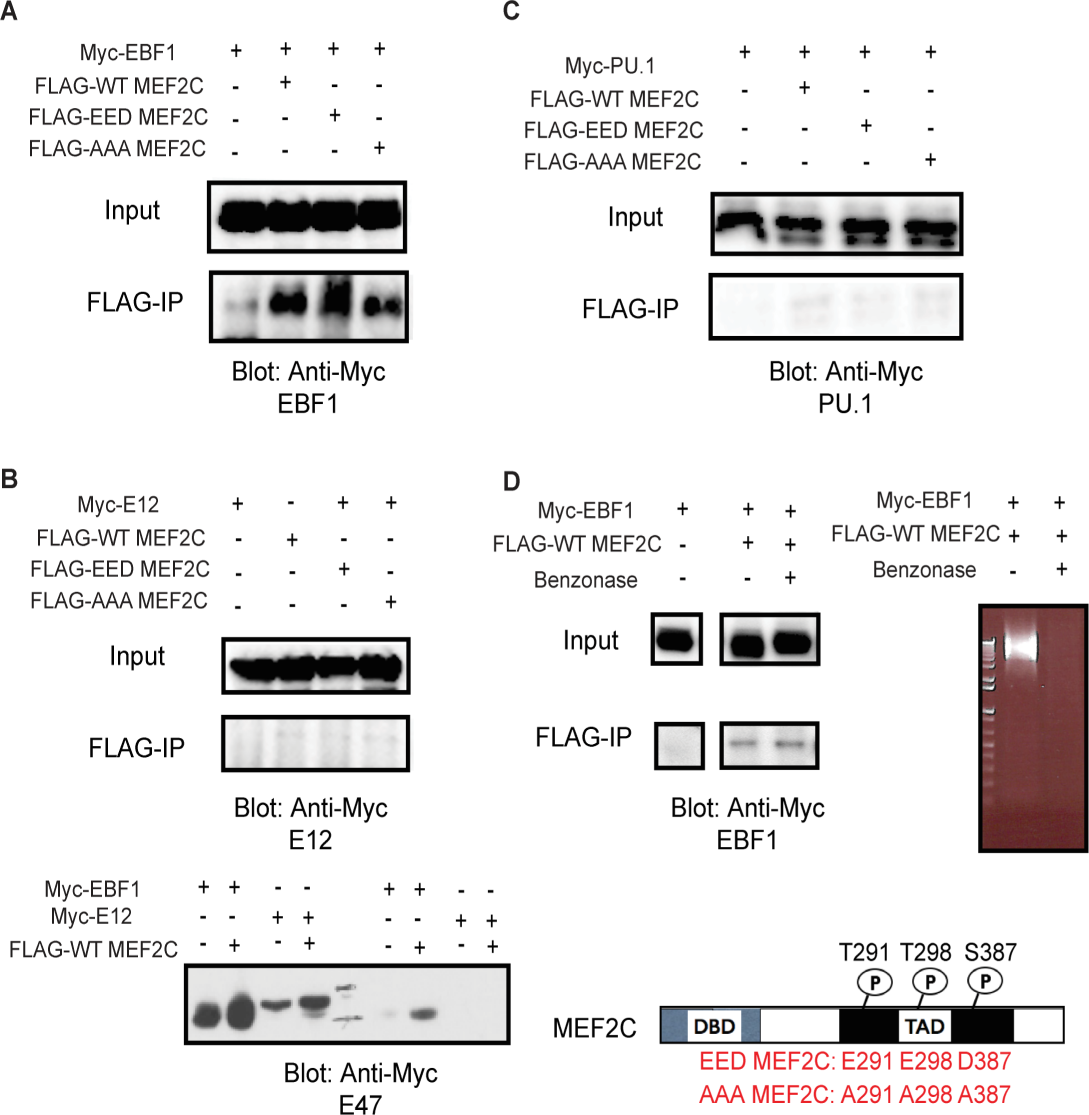
MEF2C co-immunoprecipitates with EBF1, but not other bHLH factors. FLAG-tagged WT, phosphomimetic (EED), or phosphorylation-deficient (AAA) MEF2C were co-transfected into 293T cells with Myc-tagged EBF1 (A), E12 or E47 (B), or PU.1 (C). After FLAG-IP, the immunocomplexes were blotted with anti-FLAG or anti-Myc antibodies, as indicated. (D) FLAG-tagged WT MEF2C was co-transfected into 293T cells with Myc-tagged EBF1; FLAG-IP with or without benzonase treatment and blotted with anti-Myc antibodies; the image was cropped for clarity from the same blot. Right panel: ethidium bromide gel showing all nucleic acids were degraded following benzonase treatment.

It had been shown in skeletal muscle cells that MEF2C can interact with basic helix-loop-helix (bHLH) transcription factors such as Myogenin [20]. We reasoned that it is likely MEF2C may also bind other known B cell transcription factors to direct B cell development. Mice lacking E12, PAX5 (UniProt Q02650), PU.1 (UniProt P17433), or EBF1 have defects in B cell development or reduction of early lymphoid/myeloid progenitors [21] [22] [23] [24] [25]. Therefore, we decided to test for association of MEF2C with these candidate transcription factors. FLAG-tagged MEF2C was co-transfected into 293T cells with Myc-tagged E12, E47, PAX5, PU.1, or EBF1. After FLAG pull-down, MEF2C was found to co-immunoprecipitate (co-IP) only with EBF1 but not with any other protein tested (Fig 1A, 1B and 1C, S1A and S1B Fig). This association does not appear to require DNA or RNA, as extensive digestion of nucleic acids with benzonase treatment did not prevent co-IP (Fig 1D). To examine if the phosphorylation status of MEF2C affected its association with EBF1, we generated phosphomimetic and phosphorylation-deficient MEF2C mutant proteins (EED-MEF2C and AAA MEF2C at amino acids T291, T298, and S378, respectively) and tested their ability to co-IP with EBF1 using a similar pull-down assay. Neither EED nor AAA mutants, however, showed differential abilities to co-IP with EBF1 compared to the WT MEF2C (Fig 1A, S1A and S1C Fig). Finally, co-IP after cross-linking using a human lymphoma B cell line (BJAB) and an Abelson Murine Leukemia Virus (AMuLV)-transformed B cell line (pre-B) [26] further show that MEF2C and EBF1 may exist in the same complex (S1D Fig). Thus, we have identified EBF1 as a potential novel co-regulatory partner of MEF2C.

### MEF2C and EBF1 co-occupy B lineage genes in pre-B cells

**Fig 2:**
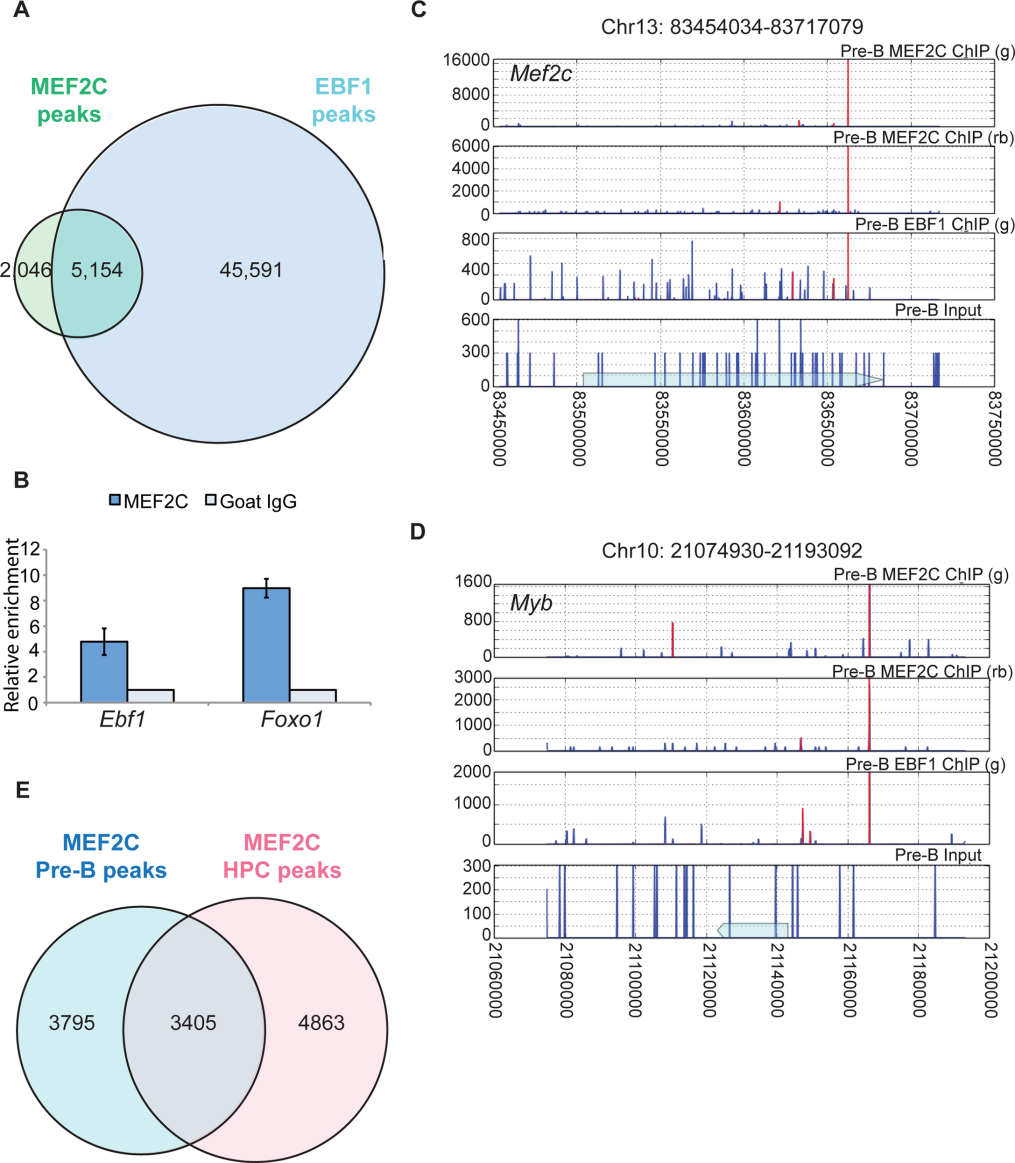
MEF2C and EBF1 co-occupy many B lineage genes. (A) Venn diagram of MEF2C and EBF1 overlapping ChIP-seq peaks in pre-B cells. MEF2C peaks are those commonly shared in two separate ChIP experiments, as shown in S2A Fig. (B) ChIP-qPCR validation of MEF2C binding near *Ebf1* and *Foxo1* genes. (C, D) Representative MEF2C and EBF1 ChIP-seq profiles at *Mef2c* and *Myb* loci, with the corresponding antibody used in the ChIP indicated (g for goat, and rb for rabbit); blue arrow on the input track indicates the position of the gene; red lines denote the highest called peak using MACS1.4. (E) Venn diagram of overlapping MEF2C ChIP-seq peaks in hematopoietic progenitor cells (HPCs) and in pre-B cells; two separate ChIP experiments were included for each cell type.

If the MEF2C and EBF1 association found in co-IP assays were important for B cell fate determination, then the two transcription factors would be expected to bind common promoters and enhancers of B cell-specific genes. To test this hypothesis, we determined whether MEF2C and EBF1 co-regulated targets in pre-B cells [26] by chromatin immunoprecipitation followed by exonuclease treatment and deep sequencing (ChIP-seq) [27]. EBF1 ChIP-seq was performed with a goat polyclonal antibody, and MEF2C ChIP-seq was performed with two different polyclonal antibodies. Subsequent comparisons were performed with overlapped peaks between the two MEF2C datasets (S2A Fig). In pre-B cells, 72% of MEF2C peaks overlapped with EBF1 peaks, which was defined as having at least 1 overlapping base pair. This comparison yielded a total of 5,154 overlapped peaks (Fig 2A), which mapped to 1,935 genes (defined as the closest genes to the peaks). In contrast, when we compared our MEF2C data set with E2A [7] or PAX5 [28] ChIP-seq data available, only 2% and 0.44% of the MEF2C peaks overlapped, respectively. Furthermore, none of the top MEF2C-bound peaks overlap with previously published PU.1 ChIP data [29]. This genome-wide analysis result is consistent with the hypothesis that MEF2C co-regulates its downstream targets with EBF1, and not other B cell-specific transcription factors. In support of their roles in B cell development, many of the top targets bound by both MEF2C and EBF1 show B cell-specific expression patterns (S1 Table). Some are key B lineage regulators such as: *Mef2c* (Entrez GeneID 17260) and *Ebf1* (GeneID 13591) themselves, *Et*s1 (GeneID 23871), *Foxo1* (GeneID 56458), and *Myb* (GeneID 17863) (representative gene tracks from ChIP-seq are shown in Fig 2C and 2D, S2C and S2D Fig). Among the targets that MEF2C and EBF1 co-regulate, *Foxo1* has previously been identified as an EBF1 target gene through ChIP-seq [7]. The finding that MEF2C directly regulates its own expression is not surprising since in skeletal muscle, a MEF2-reponsive cis-regulatory element in the *Mef2c* gene had been previously described [30]. However, we have uncovered numerous novel targets of both MFE2C and EBF1, as well as a previously unknown B cell-specific transcriptional regulatory complex. These results were validated through MEF2C and EBF1 ChIP-qPCR for selected genes (*Ebf1*, *Foxo1*, *Il7ra* (GeneID 16197), *Pou2af1* (Gene ID 18985), and *Myb*) (Fig 2B and S2B Fig). Furthermore, we confirmed the co-binding via sequential ChIP-re-ChIP of EBF1 and MEF2C (S2E Fig). To ascertain whether MEF2C can bind to its target genes prior to B cell lineage commitment, we also performed ChIP-seq using cells that over-express the transcription factor LHX2 (UniProt Q9Z0S2) and mimic hematopoietic progenitor cells (HPCs) *in vivo* and *in vitro* [31]. 41% of MEF2C peaks in HPCs overlapped with its peaks in pre-B cells, and vice versa, 47.3% of MEF2C peaks in pre-B cells were found to overlap with its peaks in HPCs, resulting in a total of 3,405 overlapping peaks (Fig 2E). *Ebf1*, *Myb*, *Foxo1*, and *Ets1* were all bound by MEF2C in HPCs (S2 Table). Interestingly, one of the MEF2C targets in both HPCs and pre-B cells is *Bach2* (Gene ID 12014), which encodes a transcriptional repressor that was recently reported to inhibit myeloid gene expression in CLPs [32]. Since EBF1 is not expressed in HPCs, these results are consistent with the notion that MEF2C binds and activates the B cell-specific transcription program during the earliest stages of commitment to the lymphoid lineage. EBF1 may then enhance transcription of some of these genes and also activate other B-lineage genes to drive B cell differentiation.

### MEF2C and EBF1 can functionally co-regulate transcription

**Fig 3:**
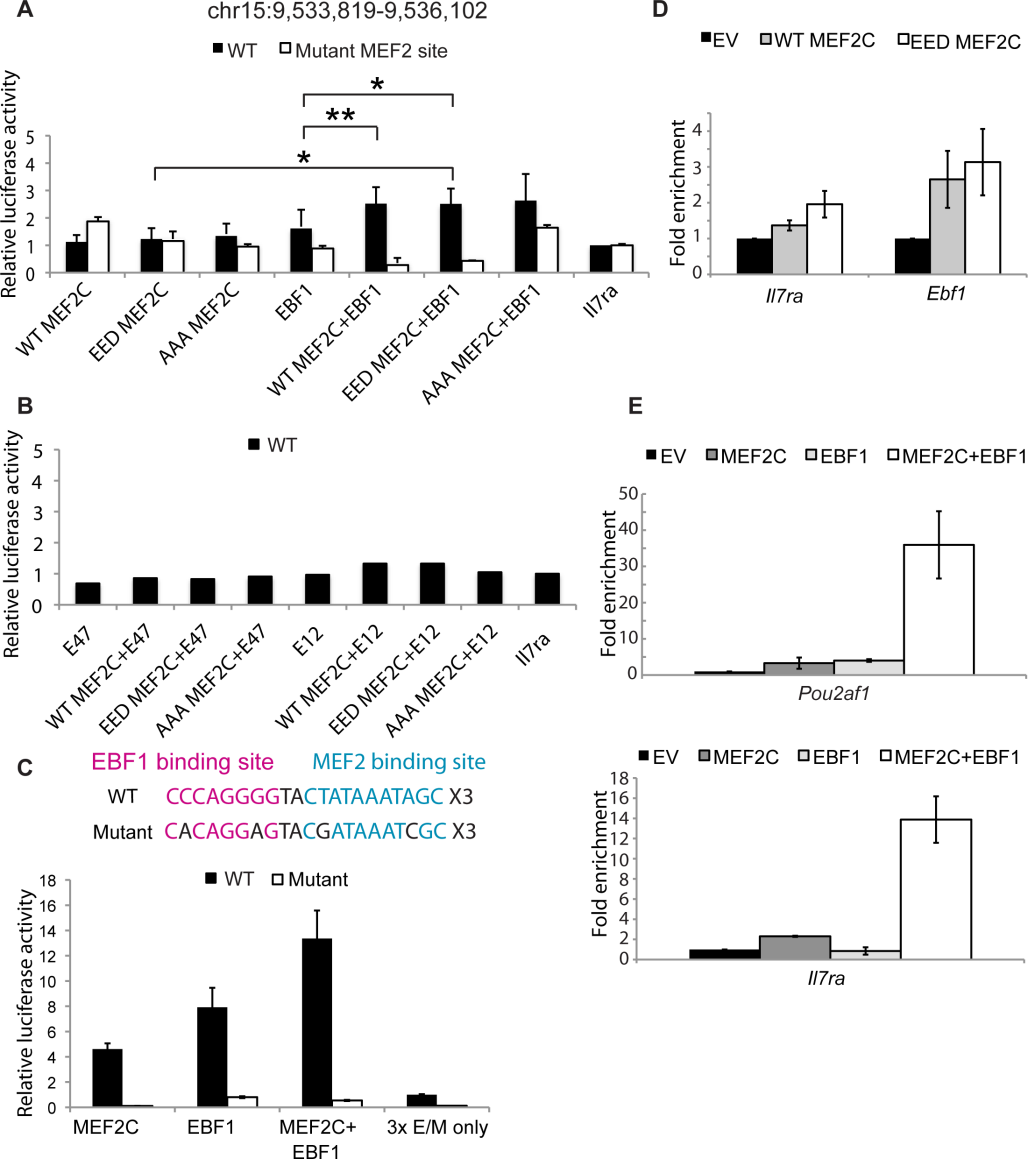
MEF2C and EBF1 can functionally co-regulate their common targets. (A) Relative luciferase activities of either WT pGL4.23-*Il7ra* (black bars) or pGL4.23-*Il7ra* containing mutated MEF2C binding site (white bars) in 293T cell lysates expressing FLAG-tagged WT, EED, or AAA MEF2C, and/or Myc-tagged EBF1, as indicated on the x-axis. Renilla luciferase was used as internal control, and the experiments were performed in biological triplicates, each with technical triplicates; asterisk denotes p-value<0.5 and double-asterisk denotes p-value<0.01 (student t-test, one-tail, paired). (B) Relative luciferase activities of WT pGL4.23-*Il7ra* in 293T cell lysates expressing the same MEF2C constructs as (A), along with Myc-tagged E12 or E47; experiments were performed in technical triplicates. (C) Luciferase reporter activities of 293T cell lysates transfected with pGL4.23-trimerized EBF1 and MEF2C binding sites with either MEF2C or EBF1 alone or in combination. Cells were transfected with either WT (WT_3xE/M, black bars) construct or a construct with mutated EBF1 and MEF2C binding sites (Mutant_3xE/M, white bars); the mutations are indicated in black in the scheme; experiment was performed in technical triplicates. (D) Relative expression levels of *Il7ra* and *Ebf1* in mouse lineage-depleted progenitor (lin-) cells that over-express either empty vector (EV), WT, or EED MEF2C, two days after transduction; summary of two biological duplicates is shown. (E) Relative expression levels of *Il7ra* and *Pou2af1* in lin- cells that over-express either EV, WT MEF2C, EBF1 individually, or MEF2C and EBF1 incombination. Summary of two biological duplicates is shown.

To functionally verify that MEF2C and EBF1 can co-regulate their target genes, targets from the pre-B ChIP-seq datasets were selected for luciferase reporter activity assays. We chose *Il7ra*, *Ebf1*, and *Foxo1*. Their regulatory regions are highly conserved between human and mouse, and contain both MEF2C and EBF1 binding sites or half sites [33] (S3 Table). *Il7ra* was previously reported as a MEF2C target and we were able to confirm the binding via ChIP-qPCR (S2B Fig). We therefore cloned the region from ChIP-qPCR analyses into a luciferase reporter vector with a minimal promoter (*Il7ra*-reporter) and tested for *Il7ra* enhancer-driven transcription activity. WT MEF2C, phosphorylation-mimetic or deficient MEF2C, and EBF1 expressing plasmids were either individually transfected or co-transfected along with the *Il7ra*-reporter. Although it has been reported that MEF2C alone can activate a synthetic luciferase reporter containing its consensus binding sites, the *Il7ra*-reporter showed only a modest activation when MEF2C and its phosphorylation site mutants were individually transfected into the cells (1.2~1.6 fold above *Il7ra*-reporter alone, Fig 3A), suggesting that there may be repressive sequences in the enhancer that counteract MEF2C’s activating function. Consistent with co-regulation by MEF2C and EBF1, the *Il7ra*-reporter showed increased luciferase activity when MEF2C and EBF1 were co-transfected, compared to either MEF2C or EBF1 alone (Fig 3A, black bars). Furthermore, when the MEF2C binding site was mutated in the *Il7ra* enhancer, the mutant reporter no longer showed MEF2C/EBF1-dependent luciferase activity (Fig 3A, white bars). In fact, the mutant reporter showed a lower activity when both MEF2C and EBF1 were transfected than either factor alone, suggesting that the cooperation between the two factors is strongly dependent on MEF2C’s ability to bind DNA. Interestingly, the difference in activities between WT and mutant templates were the most pronounced when both MEF2C (WT or EED, but not AAA mutant) and EBF1 were co-transfected. This co-regulation of the *Il7ra* -reporter between MEF2C and another transcription factor was specific to EBF1, since co-transfection of either E12 or E47 with MEF2C failed to activate the *Il7ra*-reporter (Fig 3B). This result was not due to large protein expression differences of the transfected plasmids (S3A Fig). Mutating the MEF2C phosphorylation sites did not change the luciferase result, suggesting that co-regulation with EBF1 is not kinase-dependent.

The MEF2C/EBF1 binding sites near the *Ebf1* and *Foxo1* genes also showed specific activity that was the highest when both MEF2C and EBF1 were introduced into the cells (S3B and S3C Fig). In this case, however, the relatively lower activities for co-regulation compared to either transcription factor alone is likely due to less MEF2C WT and EED protein expression in cells that were co-transfected with EBF1 (S3B Fig). As the cloned enhancers were over 600bp long, we made a synthetic luciferase reporter wherein the EBF1 and MEF2C consensus binding sites were trimerized (3xE/M) in front of a minimal promoter (Fig 3C, scheme). We found that the 3xE/M enhancer was able to drive luciferase activity when WT MEF2C and EBF1 were individually transfected into the cells, which is much higher when both proteins were co-transfected (Fig 3C, black bars). When MEF2C and EBF1 binding sites were mutated, the luciferase activities were negligible (Fig 3C, white bars). Taken together, these results suggest that in hematopoietic differentiation, MEF2C and the well-characterized B cell transcription factor EBF1 can directly activate B lineage gene transcription.

### MEF2C expression is sufficient to turn on B cell genes in hematopoietic progenitors

To further confirm that MEF2C alone can activate some of its target genes before EBF1 is expressed, we introduced either WT or EED MEF2C into lineage-depleted progenitor cells (S3E Fig) and analyzed the transcript levels of *Il7ra* and *Ebf1*. These cells were cultured under strictly undifferentiating conditions with HSC/HPC cytokines and sorted by their expression of human CD4 (hCD4) marker on the MEF2C construct two days post transduction for RNA analysis. We observed increased expression of both genes in MEF2C-expressing cells compared to empty vector (EV)-expressing cells (Fig 3D, gray and white bars compared to black bars), and EED-MEF2C expressing cells had the highest transcript levels of both genes (Fig 3D, white bars compared to gray bars). These results suggest that MEF2C is sufficient to drive key B cell-specific gene expression before other important transcription factors (i.e. EBF1) become activated during the lineage specific differentiation process.

### MEF2C and EBF1 can co-activate their target genes

To demonstrate that MEF2C and EBF1 can co-operate to activate B-cell gene expression, we introduced these two proteins either individually or in combination into progenitor cells, as described above. Cells were sorted by hCD4 for MEF2C and/or Thy1.1 or GFP for EBF1 (two separate constructs were used), and then RNA expression analyses were performed. In addition to activating their target genes *Il7ra* and *Pou2af1* alone (Fig 3E, gray bars compared to black bars), we observed a significant co-activation when both MEF2C and EBF1 were over-expressed (Fig 3E, white bars). These results suggest that the two transcription factors can genetically co-operate to activate B-cell specific genes that they co-bind.

#### MEF2C target genes in B cells have reduced expression in Mef2c-KO mice

**Fig 4:**
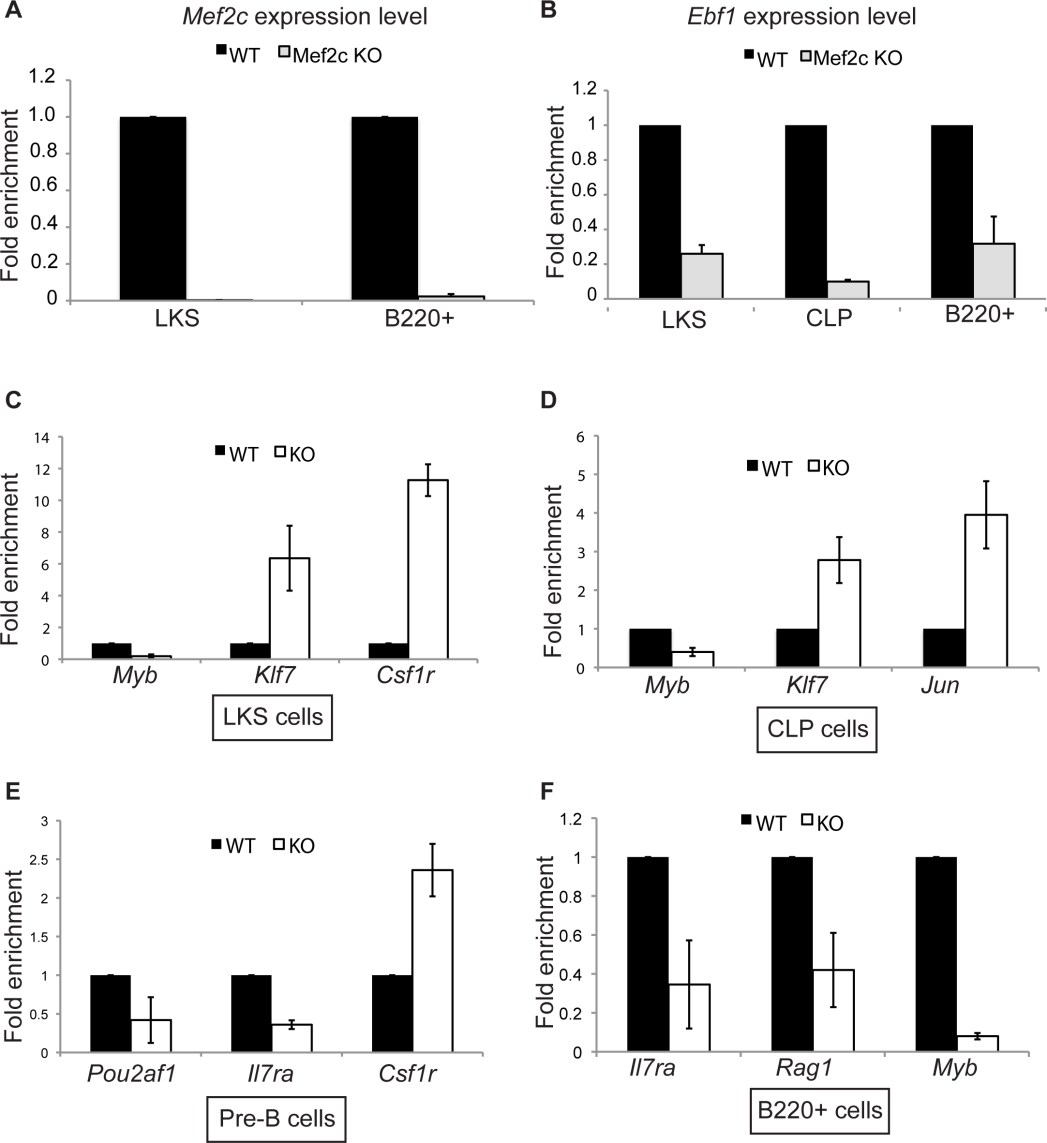
Relative expression levels of various hematopoietic genes in different lineages. (A) Relative expression levels of *Mef2c* in LKS and B220+ (all B lineage) cells sorted from *Mef2c*-KO mice (gray bars) or WT littermates (black bars). (B) Relative expression levels of *Ebf1* in LKS, CLPs, and B220+ cells sorted from same mice as in (A). (C)-(F) Relative expression levels of various B cell genes (*Myb*, *Pou2af1*, *Il7r*, and *Rag1*) and myeloid genes (*Klf7*, *Csf1r*, and *Jun*) in LKS, CLPs, Pre-B, or B220+ cells sorted from *Mef2c*-KO mice (white bars) or WT littermates (black bars) as determined by qRT-PCR. Experiments were performed in either technical triplicates or biological triplicates.

ChIP and luciferase assays suggest that MEF2C and EBF1 co-regulate their B cell-specific target genes. We, therefore, reasoned that if MEF2C functions to activate lymphoid-specific genes and repress myeloid genes, then MEF2C-null cells should have reduced lymphoid transcripts and higher myeloid gene expression. To test this hypothesis, we generated *Mef2c*-KO mice by crossing *Mx1*-Cre mice with *Mef2c*^fl/fl^ mice [12]. These animals have been reported to have decreased level of CLPs, which give rise to B, T, and natural killer (NK) cells, as well as decreased B cell differentiation potential when progenitors from the mice are cultured *in vitro* [15]. To confirm this, we isolated bone marrow from either *Mef2c*-KO or WT littermates and checked for *Mef2c* deletion as well as levels of various hematopoietic cell types. As expected, *Mef2c* transcripts were dramatically reduced in KO cells (Fig 4A) and percentages of CLPs were decreased as previously reported (S4A Fig). Additionally, percentages of B cells (B220+) were also reduced in *Mef2c*-KO mice, consistent with its known function in B cell development (S4B Fig). Interestingly, percentages of lineage negative, c-Kit+, Sca1+ (LKS) hematopoietic stem/progenitor cells were increased in KO mice (S4C Fig), suggesting that in the absence of MEF2C, mice had a blockage in stem cell differentiation and differentiation into the lymphoid lineage.

RNA-seq of *Mef2c*-KO and WT CLPs revealed that without MEF2C, many of its target genes as defined by the ChIP-seq experiments have significantly reduced expression in *Mef2c*-KO cells, especially those encoding transcription factors associated with the B lineage, such as *Ebf1*, *Bach2*, and *Ets1* (S4D Fig). Furthermore, many B cell markers or genes important for B cell functions were down-regulated in *Mef2c*-KO CLPs, including *Il12a* (Gene ID 16159), *Vpreb1* (Gene ID 22362), and *Igll1* (Gene ID 16136) (S4D Fig). Select targets from RNA-seq data and known B cell genes were further analyzed via qRT-PCR of various lineages in the blood. The B cell-specific genes—*Ebf1*, *Pou2af1*, *Myb*, *Rag1* (Gene ID 19373), and *Il7ra—*were all down-regulated in *Mef2c*-KO mice in all lymphoid lineages examined (Fig 4B to 4F). Among the down-regulated genes in *Mef2c*-KO cells, *Pou2af1* (encoding OcaB), *Vpreb1*, *Foxo1*, and *Pax5* (GeneID 18507) had previously been identified as EBF1 target genes in CLPs and different B cell stages [7,34], further supporting an important role for MEF2C in driving B cell-specific transcription together with EBF1. Consistent with the function of MEF2C as a repressor of myeloid lineage differentiation and transcription [15], sorted lymphoid cells from *Mef2c*-KO mice (CLPs and pre-B cells) have high expression levels of myeloid genes in *Mef2c*-KO mice such as *Klf7* (Gene ID 93691), *Jun* (Gene ID 16476), *Csf3r* (Gene ID 12986), *Epor* (Gene ID 13857), *Mpo* (Gene ID 17523), and *Cxcl9* (Gene ID 17329) (Fig 4D and 4E, S4D Fig). Importantly, this expression pattern was established early in LKS progenitor cells where MEF2C was first deleted in these mice (Fig 4C).

### p38 MAPK activates MEF2C in B cell differentiation

While the results above demonstrate that MEF2C co-regulate lymphoid genes with EBF1, it was still unclear how MEF2C itself is activated in this lineage-specific way. Though phosphorylation at Thr298/300 and Ser300 was dispensable for MEF2C’s ability to co-IP with EBF1 (Fig 1A), changing these residues to alanines affected the ability of MEF2C to co-regulate downstream genes with EBF1 (Fig 3A and 3D). MAPK pathways have been shown to activate MEF2 family members. Specifically, p38 MAPK was found to phosphorylate and activate MEF2C both during inflammation [17] and downstream of B cell receptor signaling to induce B cell proliferation [16]. Additionally, the extracellular signal-induced kinase 5 (ERK5) (UniProt Q9WVS8) pathway was reported to have overlapping activities with p38 MAPK at Serine 387 of MEF2C [35], which is important for apoptosis of immature T cells [36]. To determine the mechanism by which MEF2C drives progenitors to differentiate into B cells, we set up an *in vitro* differentiation assay that drives multipotent progenitors to become B cells.

**Fig 5:**
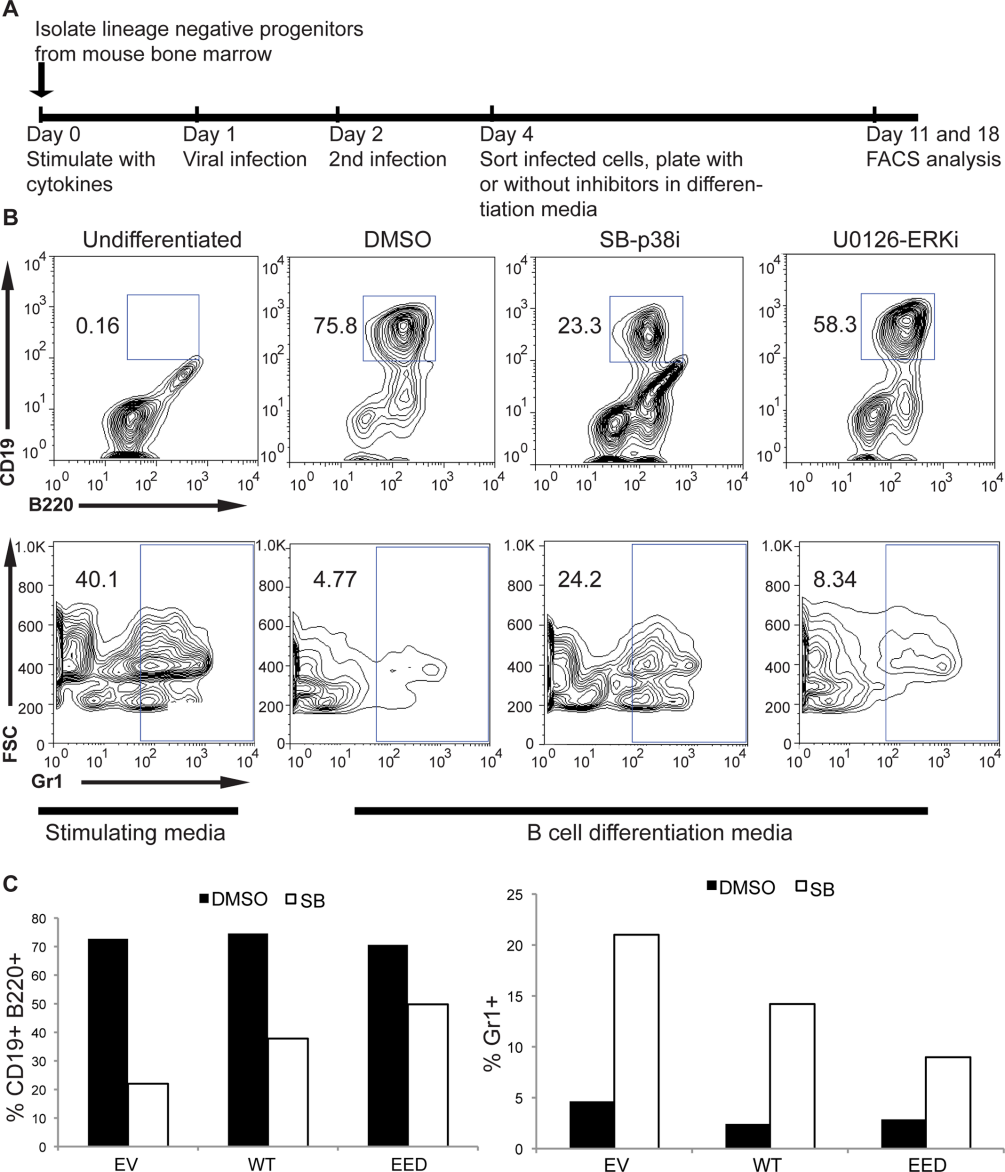
*In vitro* B cell differentiation of lineage negative progenitor cells (lin-). (A) Schematic for differentiation experiments. (B) Representative FACS plots of undifferentiated lin- cells and of those differentiated for 14 days; top panel—B220 and CD19 cell surface markers; bottom panel—forward scatter and Gr1 cell surface marker; undifferentiated cells were cultured in stimulation media; B cell differentiation cells were either untreated (DMSO), or treated with p38 MAPK inhibitor (SB-p38i), or ERK1/2/5 inhibitor (U0126-ERKi); the percentages of CD19, B220, or Gr1-positive cells are indicated. (C) Representative result of day 14 B cell differentiation of cells over-expressing empty vector (EV), WT, or EED MEF2C, treated with DMSO or SB-p38i; differentiation was measured by the expression of cell surface markers B220 and CD19 (left) or Gr1 (right). Two separate experiments were performed with similar results.

To determine the pathway responsible for activating MEF2C during lymphoid lineage specification, lineage negative (lin-) cells were treated with small molecule inhibitors targeting either p38 MAPK (SB203580, PubChem176155, or p38i) or ERK5 (U0216, PubChem3006531, or ERKi) (Fig 5A without viral infection). SB-p38i-treated cells mimicked the phenotype of *Mef2c*-KO cells, where B cell differentiation was severely impaired in comparison with DMSO control-treated cells (Fig 5B and S5A Fig, top panels). In addition, p38i-treated cells cultured in B cell-inducing media express more myeloid surface marker Gr1 (Fig 5B and S5A Fig, bottom panels). This is consistent with our hypothesized MEF2C function as a potent lymphoid fate inducer and myeloid fate repressor. Conversely, ERKi-treated cells did not show any decrease of differentiated B cells in culture (Fig 5B, S5A and S5B Fig). Cellular proliferation rates were not affected upon the treatment of either drug. Therefore, inhibiting the p38 MAPK pathway results in a B cell differentiation phenotype that is similar to deleting MEF2C in the murine blood compartment.

To confirm that the lack of MEF2C phosphorylation is responsible for the p38i phenotype, WT and EED MEF2C constructs were introduced into lin- cells prior to drug treatment. After sorting, MEF2C-expressing cells (40–65% positive for infection marker) were cultured in the same B cell differentiation condition with or without drug treatments as described above (Fig 5A). EED MEF2C-expressing cells were able to mostly overcome the B cell differentiation defect induced by p38i, whereas WT MEF2C could not (Fig 5C and S6 Fig). Analysis of myeloid differentiation, as measured by Gr1 cell surface marker, showed that by day 14, p38i-treated EV, or WT MEF2C-expressing cells all had high expression of the myeloid marker, confirming the importance of p38 MAPK in lymphoid lineage determination (Fig 5C, right panel). However, EED MEF2C-expressing cells maintained their potential to repress myeloid differentiation fate, bypassing the need for p38 MAPK (Fig 5C, right panel). Again, cell proliferation rates were unaffected by either viral infection or drug treatment. Taken together, these results suggest that MEF2C is activated through phosphorylation by the p38 MAPK pathway to drive B cell specific differentiation.

### MEF2C phosphorylation does not affect its subcellular localization

Since phosphorylation can affect the subcellular location of transcription factors [37], we tested the effect of inhibiting p38 MAPK on the nuclear localization of MEF2C. A construct containing MEF2C fused to GFP was transfected into 293T cells before treatment with either DMSO or p38i. Fluorescence images taken with DAPI nuclear stain show that MEF2C nuclear location was not affected by p38i treatment (S7A Fig). In addition, EED MEF2C did not show cytoplasmic localization in the presence of p38i (S7B Fig, left and middle panels). Finally, AAA MEF2C also showed exclusive nuclear staining (S7B Fig, right panel). These results demonstrate that phosphorylation at the three residues in MEF2C’s transactivation domain does not affect its subcellular localization.

### MEF2C may regulate the myeloid transcription program in progenitor cells through association with HDAC7

Gene expression analyses via qRT-PCR demonstrated that without MEF2C, myeloid targets were de-repressed in several blood cell types (Fig 4C to 4E). This observation was consistent with transcriptome analyses of *Mef2c*-null CLPs compared to WT cells, which showed that many myeloid genes showed up-regulated expression levels upon MEF2C depletion (S4D Fig).

Recently it has been shown that in a pre-B cell line that can be induced to trans-differentiate into myeloid cells, MEF2C associates with a class II HDAC, HDAC7, and deters the trans-differentiation of B cells to myeloid cells by repressing myeloid genes [38]. We tested this interaction by over-expressing FLAG-tagged WT MEF2C and V5-tagged murine HDAC7 in 293T cells and performed co-IP experiments. MEF2C was able to co-IP with HDAC7, consistent with previous findings (S8A Fig). This result suggests that MEF2C potentially has direct transcriptional repressive functions through associating with HDAC7, and the relief of this repression is likely important for the lymphoid versus myeloid fate specification.

Collectively, these findings help identify important players in an intricate lineage specification process and extend our understanding of the mechanism by which MEF2C drives lymphoid-specific differentiation (S8B Fig). Our working model proposes that prior to lymphoid commitment, MEF2C binds and activates genes encoding key B cell factors such as EBF1 and Bach2, the latter can directly repress myeloid transcription. After B cell specification, MEF2C and EBF1 co-regulate B cell specific genes, and many MEF2C targets have been identified here for the first time. In addition, we determined that p38 MAPK is likely the signaling pathway through which MEF2C is phosphorylated and activated during B cell differentiation.

## Discussion

How MEF2C activates lymphoid genes had not been satisfactorily answered, and the identities of most MEF2C targets were not known. This study identified a novel co-regulatory partner for MEF2C, EBF1, as well as numerous new direct targets of MEF2C, many of which encode transcription factors that were previously shown to be dependent on EBF1 expression, such as *Myb*, *Foxo1*, and *Ets1*. We have also provided genetic evidence for the sufficiency of MEF2C and EBF1 to drive B-cell gene expression in uncommitted progenitor cells. In addition, we elucidated the mechanism and pathway through which MEF2C is activated during B lineage specification. MEF2C is known to be directly activated by an Ets-family transcription factor, PU.1 [15], which is indispensable for multi-lineage hematopoietic differentiation [39]. PU.1-deficient HPCs fail to express *Il7ra* [40], which we reported here to be a direct transcriptional target of MEF2C and co-regulated by EBF1. Both PU.1 and MEF2C-deficient mice have impaired B cell specification, which may be due to failure of Interleukin-7 signaling to induce cell proliferation and drive B cell differentiation. These results place MEF2C at the top of an intricate network that is important for the specification of B cell lineage. Furthermore, a previous report that utilized a 4x MEF2C-responsive enhancer in luciferase reporter assays showed that the phosphorylation-deficient MEF2C (AAA) was unable to induce luciferase reporter activity [16]. Our experiments with native enhancers did not show a large defect for AAA MEF2C in transactivation; it was able to co-activate WT *Il7ra* luciferase activity with EBF1 as well as the WT or the phosphomimetic MEF2C (EED). However, when the MEF2C-binding site in the *Il7ra*-reporter was mutated, WT and EED MEF2C both had reduced levels of activation, and the AAA mutant did not. This suggests that the AAA MEF2C’s transactivation activity on the WT *Il7ra*-reporter is likely non-specific. Unlike their class I counterparts, class II HDACs, particularly class IIa HDAC 4, 5, 7, and 9, show tissue-specificity. *Hdac7* KO mice die early in embryogenesis due to the disruption of their endothelial cell-cell adhesion and subsequent rupture of blood vessels [41], similar to *Mef2c* conventional KO mice [42]. Direct interactions between class II HDACs and MEF2 proteins have been shown in muscle differentiation [19] and B cell lineage protection [38], suggesting a general mechanism whereby class IIa HDACs bind MEF2 family members to co-repress MEF2-dependent genes. Taken together, these results suggest that there may be some competition between EBF1 and HDAC7 for association with MEF2C, leading to a switch in its transcriptional function. It is also possible that MEF2C exists as a part of two separate complexes: one in which MEF2c is phosphorylated and activates lymphoid-specific genes; while the other in which MEF2C is unphosphorylated and participates in repressing the myeloid-specific transcription program to direct B cell differentiation (S8B Fig). In the future, more experiments will be necessary to further elucidate the validity of this model.

Class IIa HDACs are regulated by phosphorylation at conserved residues in their N-terminal domains by calcium/calmodulin-dependent protein kinases (CaMK). This phosphorylation leads to HDACs being shuttled out of the nucleus and anchored in the cytoplasm by 14-3-3 proteins, thus relieving their co-repression effect on MEF2C. Although several tissue developmental processes share this mechanism of regulating MEF2C target genes, the role of CaMK in directing lymphoid and B cell differentiation has not been elucidated. Although our results suggest that p38 MAPK phosphorylation of MEF2C is important for B cell lineage specification, it cannot be ruled out that CaMKs can also play a role in this developmental process. *Camk2d* encodes the most highly expressed CaMKll in hematopoiesis, especially at all stages of B cell development starting in pro-B cells (ImmGen database [43]). It will be of interest to treat lin- progenitor cells with CaMKII inhibitors such as KN-93 before inducing B cell development, then checking for any defects these cells might display in completing B cell differentiation.

In addition to its association with HDAC7 to directly repress myeloid transcription, MEF2C may inhibit myeloid differentiation through activating the expression of *Bach2*. BACH2 was recently shown to be a transcription repressor that binds to the Maf-recognition elements (MAREs) of several key myeloid genes such as *Cebpb* and facilitate the repression of myeloid transcription program in CLPs [32].

These findings will further contribute to a deeper understanding of the transcription network that is required to drive lymphoid and B cell-specific hematopoietic differentiation.

## Materials and Methods

### Co-immunoprecipitation

10cm plates of 293T cells were transfected using FuGene6 (Roche) with different combinations of pCMV5a-MEF2C (WT, EED, AAA)-FLAG, pCAG-EBF1-Myc and various other transcription factors as indicated, and/or pcDNA3.1-V5-HDAC7. 48 hours after transfection, cells were washed once with cold PBS and lysed with lysis buffer (50mM HEPES, 140mM NaCl, 1mM EDTA, 0.5% Triton-X, 0,5% sodium deoxycholate, and fresh protease inhibitors, PMSF, DTT, and benzamidine). The lysates were passed through a 25-gauge needle 10 times, and collected by centrifugation. IP was performed overnight with anti-FLAG M2 antibodies (Sigma), washed with high-salt lysis buffer (500mM NaCl), and the immunocomplex was boiled in SDS sample buffer for 10 minutes before SDS-PAGE and western blotting, along with saved input. ChemiDoc and ImageLab software (Bio-Rad) were used to detect and analyze the data, respectively. Endogenous IP experiments were performed with mouse pre-B cells and human BJAB cell lines that were cross-linked for 10 minutes with 1% formaldehyde. Overnight IP with MEF2C antibodies (Cell Signaling 5030, CST) or control normal rabbit IgGs (Santa Cruz sc2027) were performed, washed next day with high-salt lysis buffer, boiled and immunoblotted with EBF1 antibody (Sigma SAB2501166).

### Chromatin immunoprecipitation followed by exonuclease treatment and deep sequencing (ChIP-seq)

1x10^8^ pre-B or LHX2-HPC cells were crosslinked with 1% formaldehyde for 10 minutes. The chromatin was fragmented to 100–300bp with a water bath sonicator (Adaptive Focused Acoustics, Covaris S220). IP was performed overnight with pre-blocked antibody-bead complex with MEF2C (Santa Cruz sc-13266 or CST) or EBF1 (Sigma SAB2501166) antibodies. While the immunocomplex was still immobilized on the beads, it was subjected to a series of washes and enzymatic treatments, in order: end polishing with T4 DNA polymerase, P2 adaptor ligation (sequences were adapted from Illumina small RNA library prep kit), nick repair with phi29 polymerase, 5’ to 3’ DNA digestion with Lambda and RecJ_f_ exonucleases. After washing, the MEF2C or EBF1-immunocomplex was eluted and cross-linking was reversed at 65°C. After proteinase K and RNaseA treatments, phenol-chloroform DNA purification was performed before the libraries were constructed (llumina), quality-controlled (2100Bioanalyzer, Agilent), and sequenced (HiSeq2000, Illumina). Single-end reads were mapped with Burrows-Wheeler Aligner (BWA) to the repeat-masked mouse mm10 genome (UCSC Genome Browser). Following the removal of duplicated reads to reduce the impact of PCR duplication artifacts, peaks were called using Model-Based Analysis for ChIP-seq (MACS) 1.4 software [44] with input DNA as the control dataset. Different lineage-specific expression patterns of the targets were determined through qPCR or the ImmGen database [43]. For ChIP-qPCR, absolute percentage of input DNA pulled-down in each IP was calculated from comparison with standard dilution of the input (3 dilutions for each set of primers), then fold enrichment was calculated for each IP as compared to isotype-matched IgG.

### Luciferase reporter assays

293T cells grown in 24-well plates were transfected with different combinations of FLAG-tagged MEF2C (WT, EED, or AAA), Myc-tagged EBF1, E12, or E47, as well as pGL4.23-*Il7*, *Ebf1*, or *Foxo1* driving the *Luc2* firefly luciferase, and internal renilla luciferase (pRL-TK, Promega). Cell lysates were collected according to manufacturer’s instructions and the relative luciferase activities were measured using the Dual-Luciferase Reporter Assay kit (Promega) and either a GloMax 20/20 luminometer (Promega) or SpectraII Microplate Reader (Tecan).

### RNA extraction from small-size samples and RNA-seq

Trizol containing linear polyacrylamide (PLA, 5uL/mL of Trizol) was used to lyse the sorted cells (800–5,000). After 10-second vortex and 3-minute incubation at RT, chloroform (200uL) was added, followed by a 30-second vortex and spin at 4°C. The aqueous layer containing RNA was mixed with 0.7 volumes of isopropanol, and precipitated overnight at -20°C, then washed and re-dissolved. First-strand cDNA synthesis was performed either with iScript (Bio-Rad) or SuperScript III (Life Technologies) reverse transcriptase, and qPCR was performed with real-time PCR instruments (Applied Biosystems or Bio-Rad CFX96). Ribonucleoprotein (RNP) and glyceraldehyde 3-phosphate dehydrogenase (GAPDH) transcripts were used as internal controls. All qPCR primers are listed in S4 Table. RNA-seq libraries were constructed according to manufacturer’s instructions (Illumina), quality-controlled (2100Bioanalyzer, Agilent), and sequenced (HiSeq2000, Illumina). Reads were mapped to the UCSC mouse genome mm10 and analyzed with the Tuxedo Suite software (University of Missouri Bioinformatics Consortium) and CummeRbund software (MIT Computational Biology Group and Broad Institute). The qRT-PCR experiments were performed in biological triplicates; fold enrichment is calculated by setting expression level in WT animals as 1.

### *Mef2c*-KO mice

*Mef2c*-floxed mice (129S) [12] were mated with Mx1-Cre mice (Black6, Jackson labs). The offspring were backcrossed for >4 generations to generate either *Mef2c*^flox/flox^/Cre+ (KO), or *Mef2c*^flox/flox^/Cre- (WT) littermates. Sex-matched littermate mice with the desired genotypes were injected intraperitoneally with 400ug of synthetic polyinosinic:polycytidylic acid (pIpC) to induce Cre expression and *Mef2c* deletion. A total of four injections every other day were performed.

### Ethics statement

All animal work was conducted according to guidelines of the Association for Assessment and Accreditation of Laboratory Animal Care, and was approved by the Animal Care and Use Committee at University of California, Berkeley (protocol number R007). Mice used in this study were sacrificed by carbon dioxide asphyxiation followed by cervical dislocation.

### FACS sorting and analysis

Bone marrow was collected from femur bones of 6 to 12-week old mice, and in the case of lineage negative (lin-) cell selection, mouse lineage cell depletion kit (130-090-858, Miltenyi Biotec) was used. CLPs were sorted with cell surface makers developed by Kondo et al. [45], namely lineage negative, Sca1 low, c-Kit low, and Il7r positive. An Influx sorter (BD Biosciences) was used to sort cells, and LSRII or LSRFortessa flow cytometers (BD Biosciences) were used to collect data and FlowJo software was used to analyze the data.

### Cell culturing and lentiviral infection

Abelson Murine Leukemia Virus (AMuLV)-transformed B cells (pre-B cells) were cultured in RPMI (Gibco) with 5% FBS (Gemini) and beta-mercaptoethanol (2-ME, Gibco). LHX2-HPC cells (gift from Dr. Leif Carlsson’s lab) were maintained in IMDM (Gibco) supplemented with 10% FBS, 2-ME, human IL-3 (10ng/mL), and murine stem cell factor (SCF, 20ng/mL). Viruses were produced in 293T cells, filtered with 45uM syringe filters, and concentrated by ultra-centrifugation at 19,000rpm. Lin- cells were cultured overnight in stimulation media (100ng/mL Flt3L, 50ng/mL SCF, and 2ng/mL TPO), spin-infected twice with viruses containing polybrene (8ug/mL) at 1,000rpm for 1.5 hours at room temperature. RNA extraction and transcript analysis were performed two days after viral transduction and sorting. B cell differentiation media is the same as stimulation media without TOP but containing interleukin-7 (25ng/mL). During B cell differentiation, untreated cells had DMSO added to the media, while other cells were treated with either p38 MAPK inhibitor (SB-p38i) or ERK1/2/5 inhibitor (U0216-ERKi). MEF2C-expressing cells were sorted by IRES-hCD4 marker on the construct, and EBF1-expressing cells were sorted by either IRES-GFP or IRES-Thy1.1 markers on the constructs.

## Supporting Information

S1 Fig MEF2C does not co-immunoprecipitates with PAX5 and is pulled down at similar levels in transfected cells. (A) MEF2C input and recovery of FLAG-IP experiments in Fig 1 are similar across the different co-transfections. (B) FLAG-tagged WT or EED MEF2C co-transfected into 293T cells with Myc-tagged PAX5; FLAG-IP was then blotted with the indicated antibodies. Asterisks denote lanes from an unrelated experiment. (C) FLAG-tagged WT, EED or AAA MEF2C co-transfected into 293T cells with Myc-tagged EBF1; Myc-IP was blotted with FLAG antibody. (D) Co-IP of endogenous MEF2C and EBF1 in pre-B cells or BJAB cells after cross-linking.
(TIF)

S2 Fig MEF2C and EBF1 co-occupy many B lineage genes. (A) Venn diagram of overlapping MEF2C ChIP-seq peaks in pre-B cells from two different antibodies (Santa Cruz and Cell Signaling). (B) ChIP-qPCR validation of MEF2C binding near *Il7ra* and *Myb* genes (top) and EBF1 binding near *Foxo1*, *Il7ra*, *Pou2af1*, and *Myb* genes (bottom). (C, D) Representative MEF2C and EBF1 ChIP-seq profiles at *Ebf1* and *Pax5* loci, with the corresponding antibody used in the ChIP; blue arrow on the input track indicates the position of the gene; red lines denote the highest called peak using MACS. (E) Sequential ChIP of EBF1 and MEF2C (top) and the reverse (bottom) at several of their target genes.
(TIF)

S3 Fig Luciferase reporter assays show MEF2C and EBF1 can functionally co-regulate their common targets. (A) Relative luciferase activities of pGL4.23-*Ebf1* in 293T cell lysates transfected with FLAG-tagged WT, EED, MEF2C, and/or Myc-tagged EBF1, and Renilla luciferase internal control vector; the experiments were performed in technical triplicates. (B) Expression levels of various MEF2C and EBF1 constructs in the cell lysates used in luciferase reporter assays in (A), blotted with either anti-FLAG or anti-Myc antibodies, as indicated. The asterisk denotes a band from an unrelated experiment. (C) Relative luciferase activities of pGL4.23-*Foxo1* in 293T cell lysates expressing the same activators as (A); the experiments were performed in technical triplicates. (D) Expression levels of MEF2C and EBF1 in cell lysates used in luciferase reporter assays in Fig 3C. (E) Relative expression levels of *Mef2c* in mouse lineage-depleted progenitor cells that over-express either empty vector (EV), WT, or EED MEF2C; summary of two biological duplicates is shown.
(TIF)

S4 Fig Percentages of various hematopoietic cell types in *Mef2c*-KO mice compared to WT littermates. Percentages of common lymphoid progenitors (CLPs) in total bone marrow cells (A) and B220+ (B lineage) cells in lineage positive population (B) from 6–8 weeks-old mice with Mx1-Cre mediated deletion of *Mef2c* exon2, compared to WT littermates. The experiments were performed in biological triplicates. (C) The ratio of the percentages of lineage negative, c-Kit positive, Sca-1 positive (LKS) progenitors in *Mef2c*-KO mice compared to WT littermates. Data from four separate pairs are shown; the percentages were calculated each time either by comparing number of LKS cells in lineage negative population or in total bone marrow compartment. (D) Heat map of selected RNA-seq results from WT or *Mef2c*-KO CLPs; in black are B cell-specific genes, and in red are myeloid-specific genes.
(TIF)

S5 Fig *In vitro* B cell differentiation of lin- cells. (A) Representative FACS plots of undifferentiated lin- cells or those on day 14 of B cell differentiation, either untreated (DMSO), treated with p38i (p38 MAPK inhibitor), or U0126 (ERK inhibitor), as measured by CD19 and B220 (top panel), or myeloid marker Gr1 (bottom panel) expression. (B) Summary of drug treatment results from Fig 5B and S5A Fig.
(TIF)

S6 Fig B cell differentiation defects of p38i-treated lin- cells can be rescued by MEF2C mutant. FACS plots of summarized results from Fig 5C. Day 14 B cell differentiation of lin- cells expressing empty vector (EV) (A), WT MEF2C (B), or EED MEF2C (C), as measured by B220 and CD19 surface marker expression. (D) Summary of drug treatment and rescue results from two separate experiments. Rescue index was calculated as follows: the ratio of p38i and DMSO-treated, EV-expressing lin- cells after differentiation was set as one to represent the baseline inhibition (raw data were percentages of cells expressing both B220 and CD19 markers); then the p38i/DMSO ratio of WT or EED MEF2C-expressing cells were compared to the baseline inhibition.
(TIF)

S7 Fig MEF2C shows exclusive nuclear localization, despite its phosphorylation status. 293T cells were transiently transfected with WT MEF2C-GFP (A), EED MEF2C-GFP or AAA MEF2C-GFP (B), then cultured in either untreated condition (DMSO) or with p38 MAPK inhibitor SB203580 (p38i), except for the AAA MEF2C-transfected cells. Confocal images with DAPI nuclear staining (blue) were taken 48 hours after transfection, showing GFP (green) expression that indicates the subcellular localization of MEF2C.
(TIF)

S8 Fig MEF2C co-immunoprecipitates with HDAC7. (A) FLAG-tagged WT MEF2C was co-transfected into 293T cells with V5-tagged HDAC7; FLAG-IP was blotted with anti-V5 antibody (top portion) or anti-FLAG antibody (bottom portion). Image was cropped from the same blot for clarity. Asterisk denotes heavy chain contamination, which is slightly smaller than MEF2C. (B) Model of B cell-specific transcription and lineage determination that requires MEF2C.
(TIF)

S1 Table Examples of B cell-specific genes near MEF2C and EBF1 ChIP-seq peaks in pre-B cells. Results from two different ChIP experiments are shown here. The gene name, start, and end of each gene are bolded. The chromosome, start, end, and the score of each MACS-called peak are listed under each gene. All genes shown have binding overlap between EBF1 and both MEF2C datasets, except for the gene in parenthesis, which had binding overlap between EBF1 and only one of the MEF2C datasets.
(PDF)

S2 Table B cell-specific genes near MEF2C ChIP-seq peaks in hematopoietic progenitor cells (HPCs). Results from two different ChIP-experiments are shown here. The gene name, start, and end of each gene are bolded. The chromosome, start, end, and the score of each MACS-called peak are listed under each gene.
(PDF)

S3 Table Genomic regions used in luciferase reporter assays. Genomic sequences of murine *Il7ra*, *Ebf1*, and *Foxo1* genes that were cloned into pGL4.23 luciferase reporters are listed here. Bolded are MEF2C consensus binding sites. Underlined are potential EBF1 binding sites or half sites. Bolded and underlined are sequences that were mutated.
(PDF)

S4 Table List of qPCR primers used in this study. (PDF)
