# Supplementary figures and images for: MEF2C and EBF1 Co-regulate B Cell-Specific Transcription
Source: PLoS Genet. 2016 Feb 22;12(2):e1005845. doi: 10.1371/journal.pgen.1005845 (PMC4762780; doi:10.1371/journal.pgen.1005845)

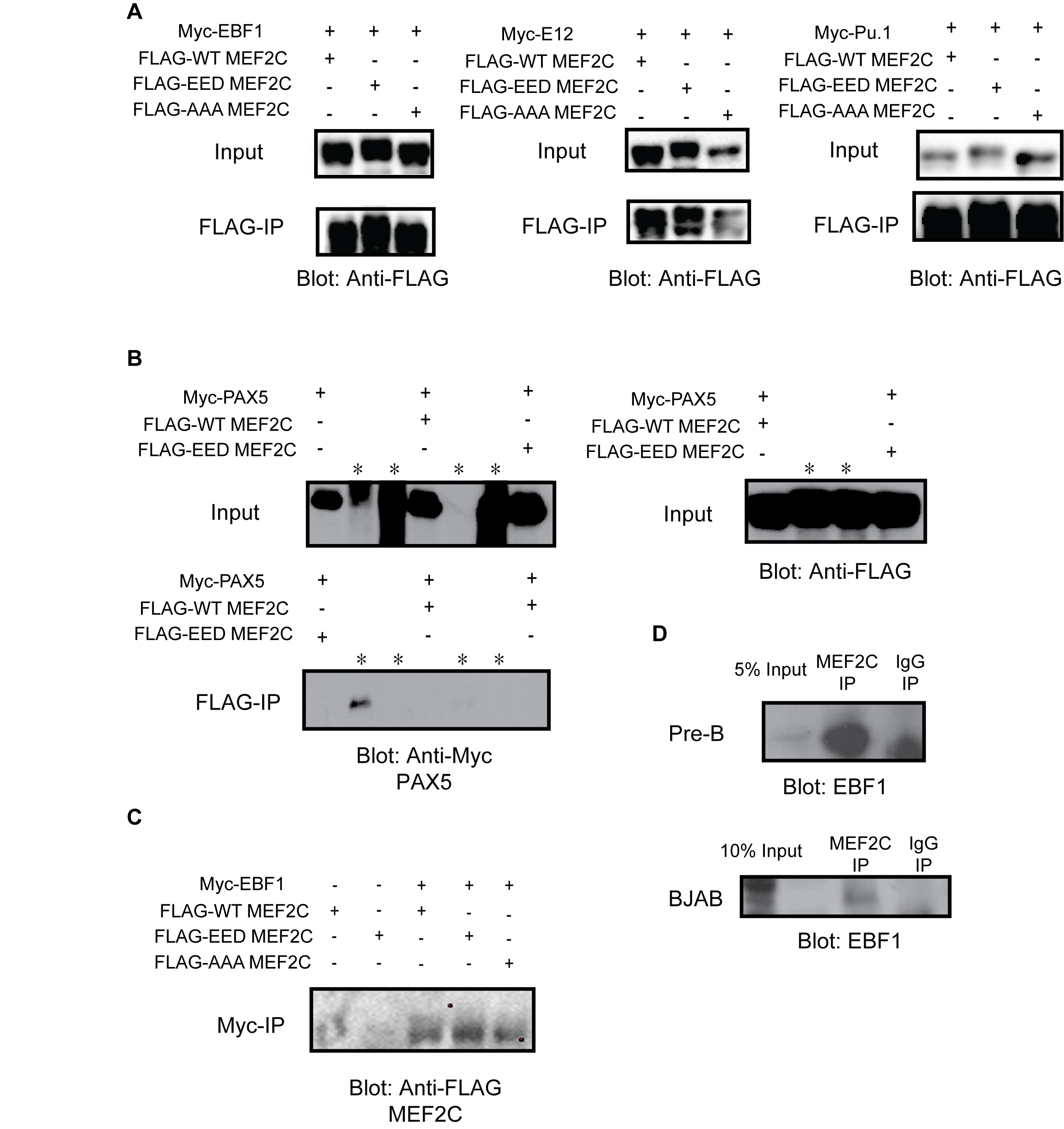

Supplement: S1 Fig — (A) MEF2C input and recovery of FLAG-IP experiments in Fig 1 are similar across the different co-transfections. (B) FLAG-tagged WT or EED MEF2C co-transfected into 293T cells with Myc-tagged PAX5; FLAG-IP was then blotted with the indicated antibodies. Asterisks denote lanes from an unrelated experiment. (C) FLAG-tagged WT, EED or AAA MEF2C co-transfected into 293T cells with Myc-tagged EBF1; Myc-IP was blotted with FLAG antibody. (D) Co-IP of endogenous MEF2C and EBF1 in pre-B cells or BJAB cells after cross-linking. (TIF) [file pgen.1005845.s001.tif]

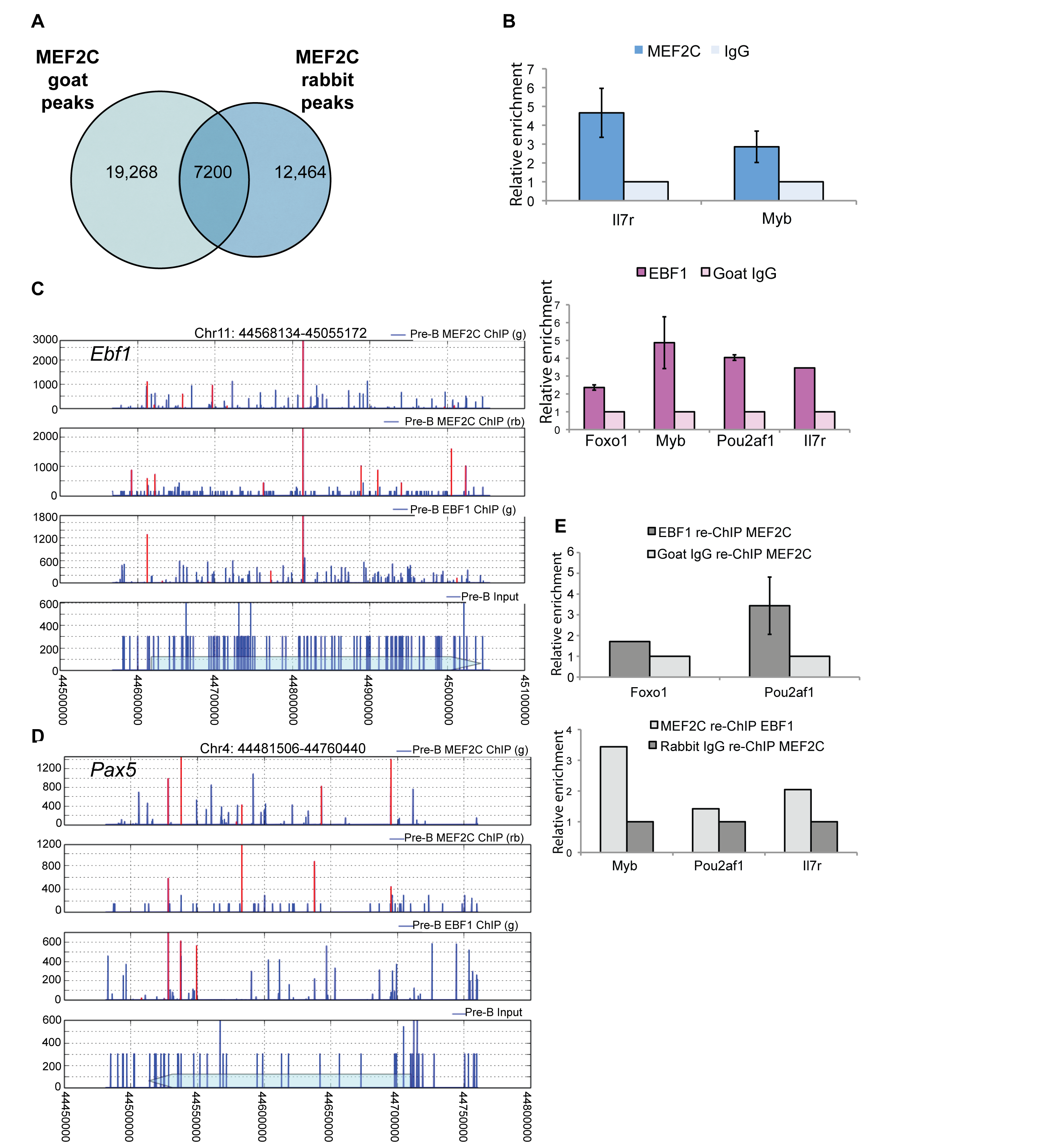

Supplement: S2 Fig — (A) Venn diagram of overlapping MEF2C ChIP-seq peaks in pre-B cells from two different antibodies (Santa Cruz and Cell Signaling). (B) ChIP-qPCR validation of MEF2C binding near Il7ra and Myb genes (top) and EBF1 binding near Foxo1, Il7ra, Pou2af1, and Myb genes (bottom). (C, D) Representative MEF2C and EBF1 ChIP-seq profiles at Ebf1 and Pax5 loci, with the corresponding antibody used in the ChIP; blue arrow on the input track indicates the position of the gene; red lines denote the highest called peak using MACS. (E) Sequential ChIP of EBF1 and MEF2C (top) and the reverse (bottom) at several of their target genes. (TIF) [file pgen.1005845.s002.tif]

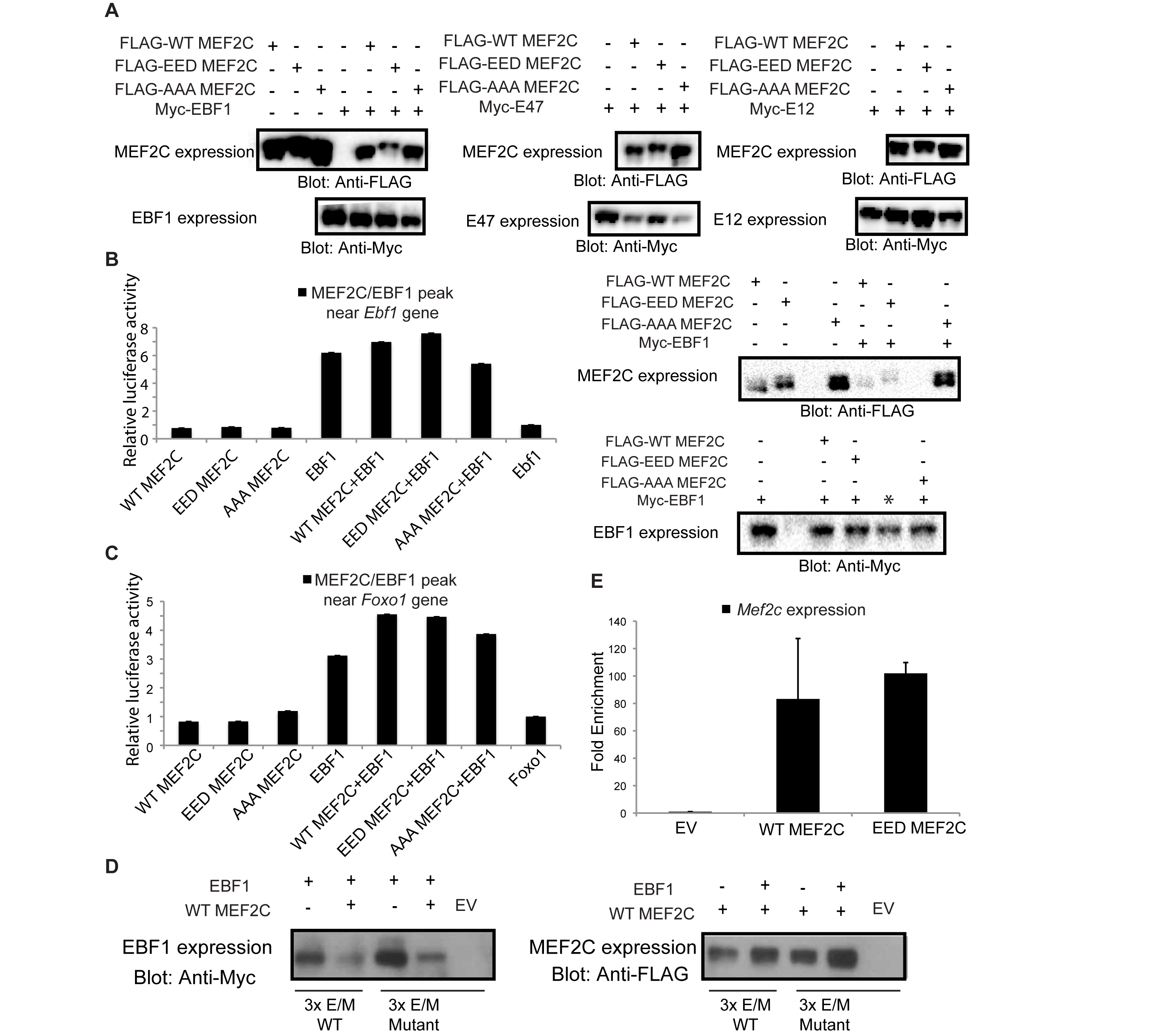

Supplement: S3 Fig — (A) Relative luciferase activities of pGL4.23-Ebf1 in 293T cell lysates transfected with FLAG-tagged WT, EED, MEF2C, and/or Myc-tagged EBF1, and Renilla luciferase internal control vector; the experiments were performed in technical triplicates. (B) Expression levels of various MEF2C and EBF1 constructs in the cell lysates used in luciferase reporter assays in (A), blotted with either anti-FLAG or anti-Myc antibodies, as indicated. The asterisk denotes a band from an unrelated experiment. (C) Relative luciferase activities of pGL4.23-Foxo1 in 293T cell lysates expressing the same activators as (A); the experiments were performed in technical triplicates. (D) Expression levels of MEF2C and EBF1 in cell lysates used in luciferase reporter assays in Fig 3C. (E) Relative expression levels of Mef2c in mouse lineage-depleted progenitor cells that over-express either empty vector (EV), WT, or EED MEF2C; summary of two biological duplicates is shown. (TIF) [file pgen.1005845.s003.tif]

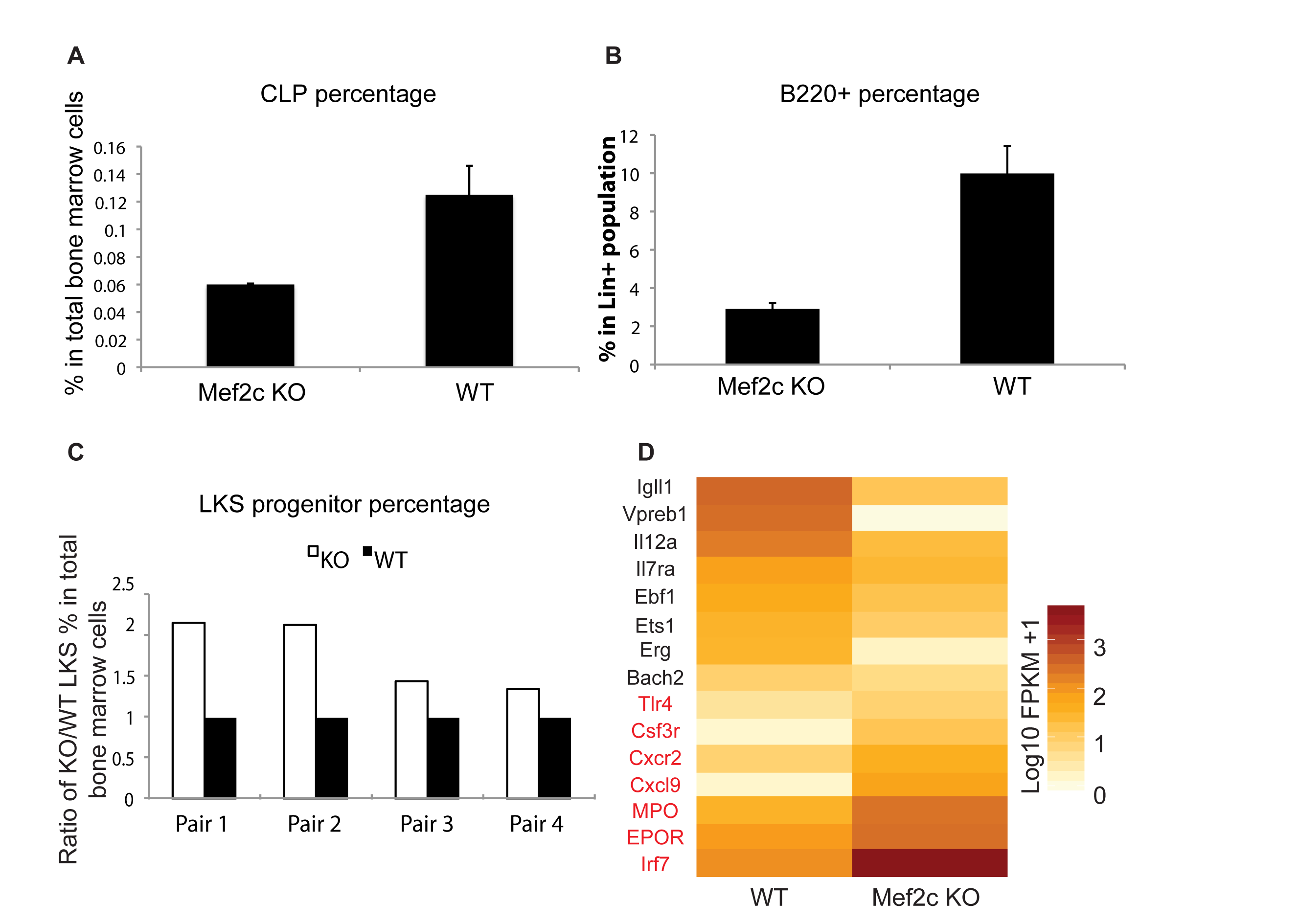

Supplement: S4 Fig — Percentages of common lymphoid progenitors (CLPs) in total bone marrow cells (A) and B220+ (B lineage) cells in lineage positive population (B) from 6–8 weeks-old mice with Mx1-Cre mediated deletion of Mef2c exon2, compared to WT littermates. The experiments were performed in biological triplicates. (C) The ratio of the percentages of lineage negative, c-Kit positive, Sca-1 positive (LKS) progenitors in Mef2c-KO mice compared to WT littermates. Data from four separate pairs are shown; the percentages were calculated each time either by comparing number of LKS cells in lineage negative population or in total bone marrow compartment. (D) Heat map of selected RNA-seq results from WT or Mef2c-KO CLPs; in black are B cell-specific genes, and in red are myeloid-specific genes. (TIF) [file pgen.1005845.s004.tif]

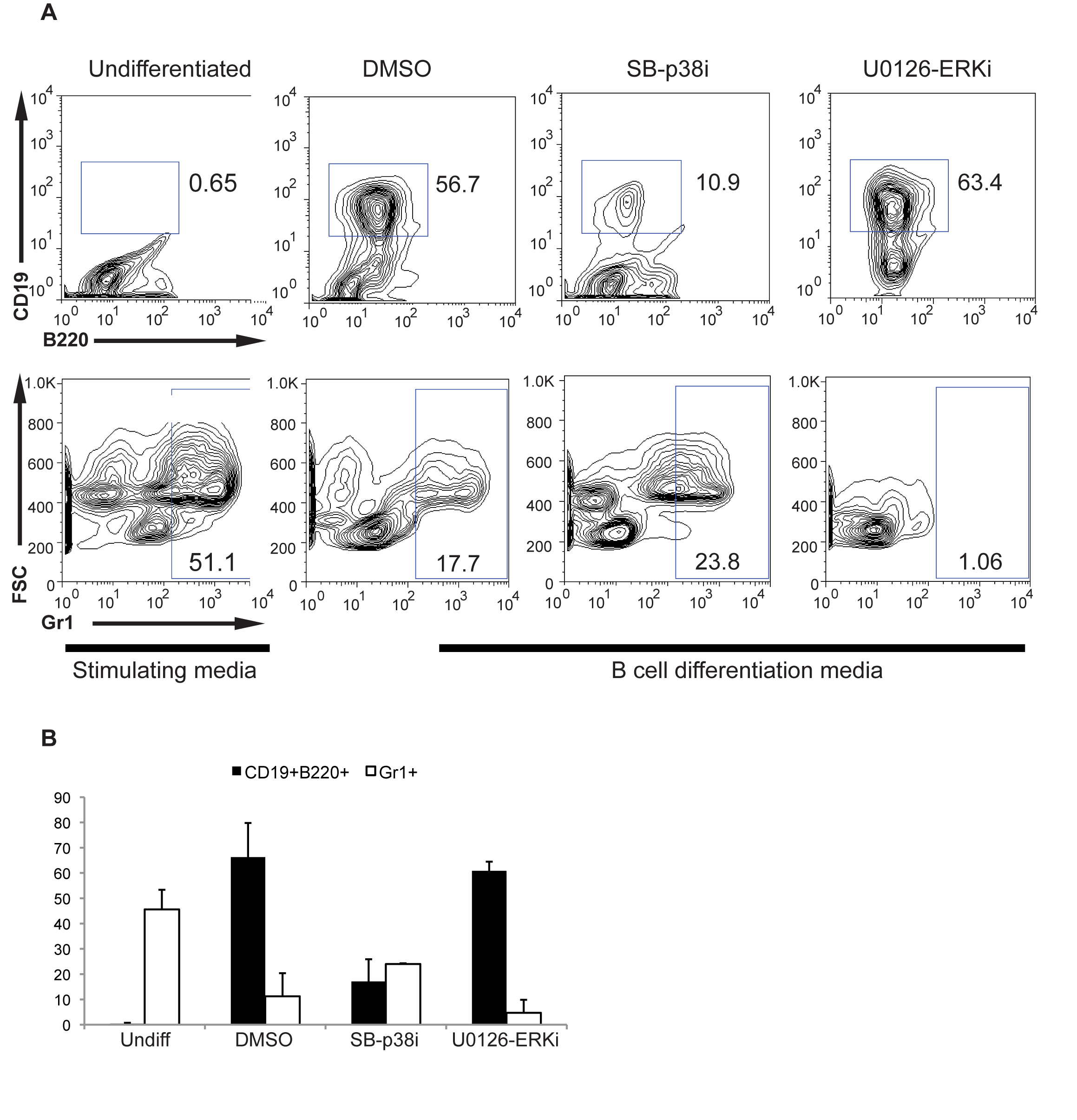

Supplement: S5 Fig — (A) Representative FACS plots of undifferentiated lin- cells or those on day 14 of B cell differentiation, either untreated (DMSO), treated with p38i (p38 MAPK inhibitor), or U0126 (ERK inhibitor), as measured by CD19 and B220 (top panel), or myeloid marker Gr1 (bottom panel) expression. (B) Summary of drug treatment results from Fig 5B and S5A Fig. (TIF) [file pgen.1005845.s005.tif]

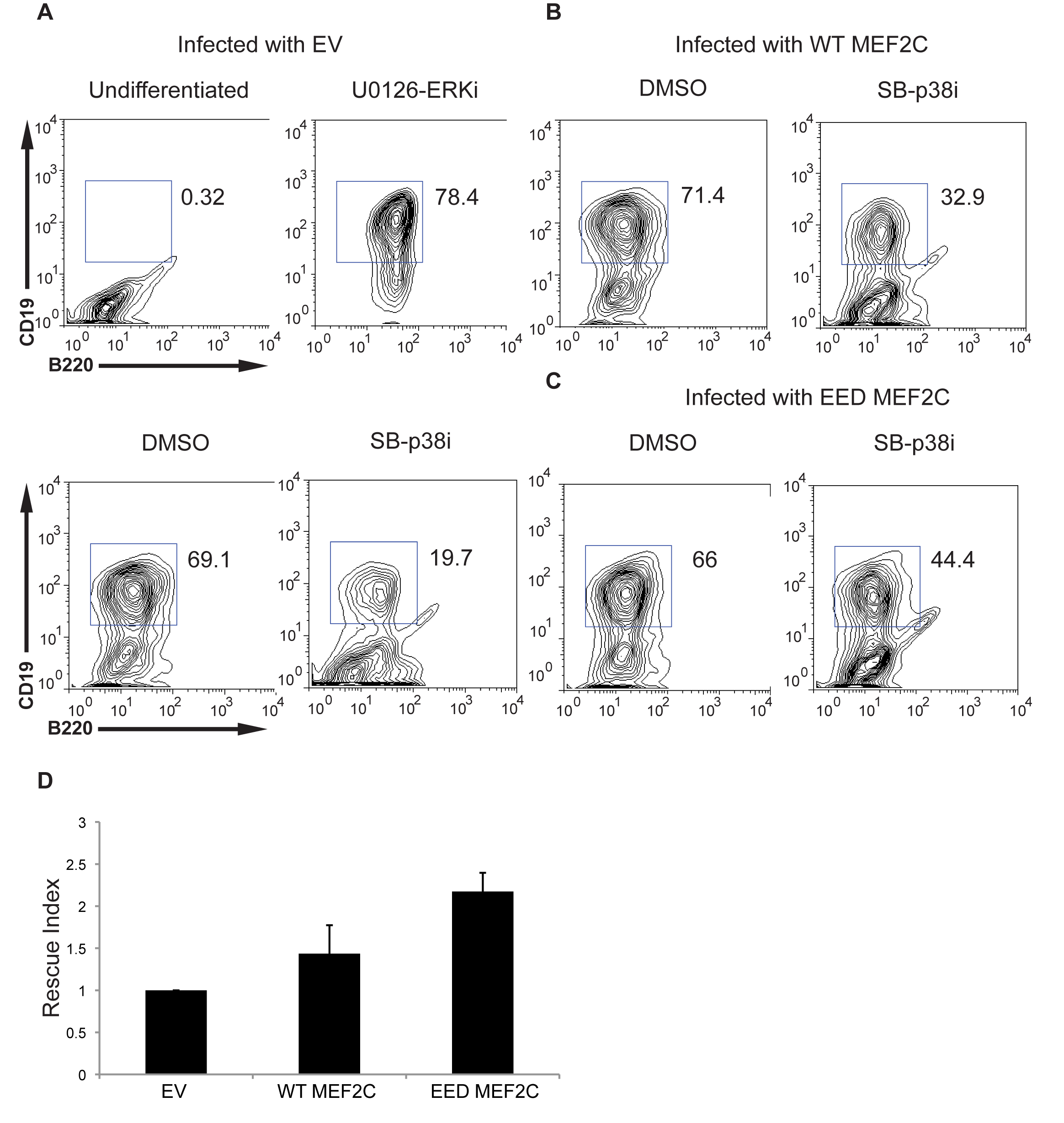

Supplement: S6 Fig — FACS plots of summarized results from Fig 5C. Day 14 B cell differentiation of lin- cells expressing empty vector (EV) (A), WT MEF2C (B), or EED MEF2C (C), as measured by B220 and CD19 surface marker expression. (D) Summary of drug treatment and rescue results from two separate experiments. Rescue index was calculated as follows: the ratio of p38i and DMSO-treated, EV-expressing lin- cells after differentiation was set as one to represent the baseline inhibition (raw data were percentages of cells expressing both B220 and CD19 markers); then the p38i/DMSO ratio of WT or EED MEF2C-expressing cells were compared to the baseline inhibition. (TIF) [file pgen.1005845.s006.tif]

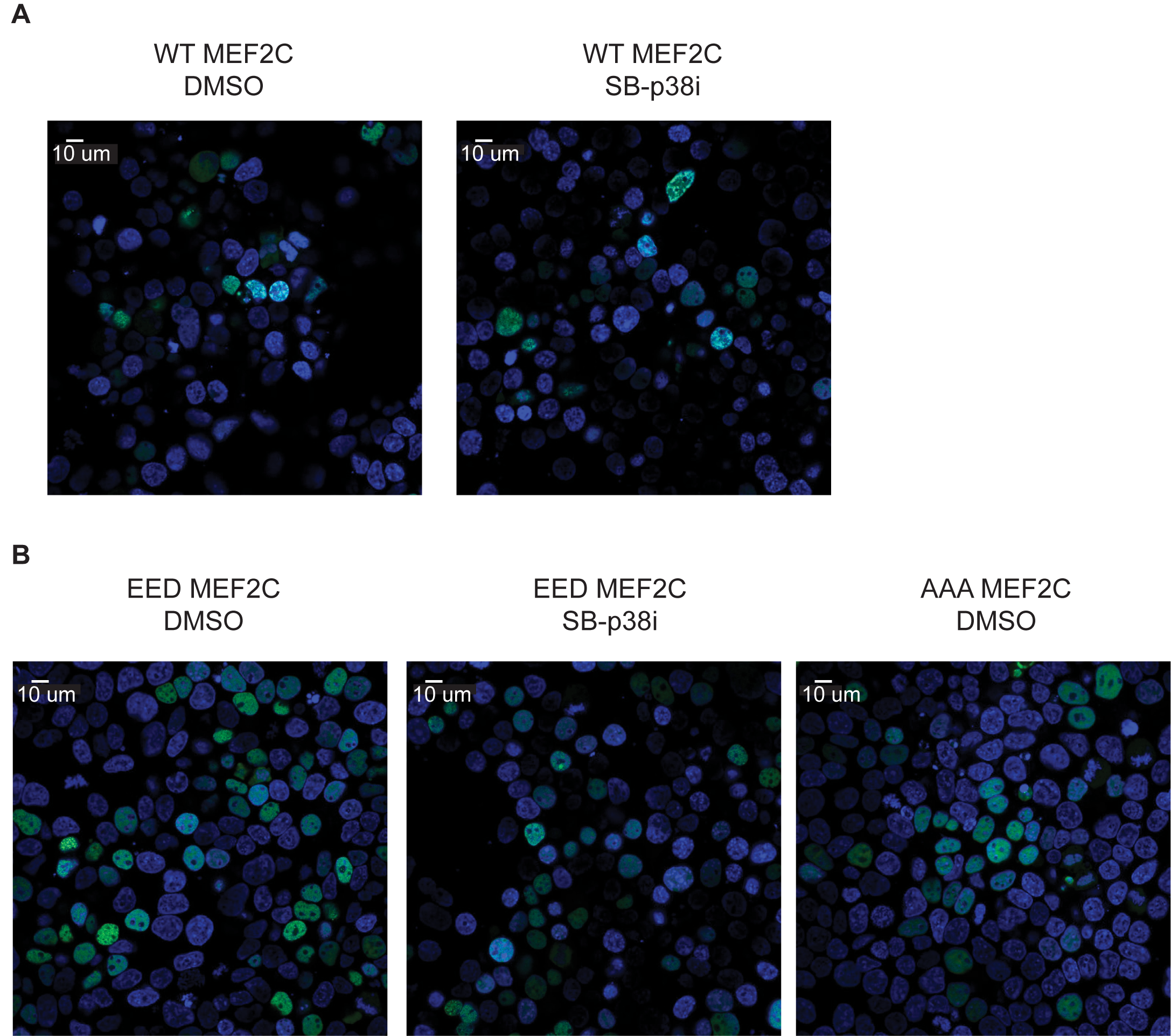

Supplement: S7 Fig — 293T cells were transiently transfected with WT MEF2C-GFP (A), EED MEF2C-GFP or AAA MEF2C-GFP (B), then cultured in either untreated condition (DMSO) or with p38 MAPK inhibitor SB203580 (p38i), except for the AAA MEF2C-transfected cells. Confocal images with DAPI nuclear staining (blue) were taken 48 hours after transfection, showing GFP (green) expression that indicates the subcellular localization of MEF2C. (TIF) [file pgen.1005845.s007.tif]

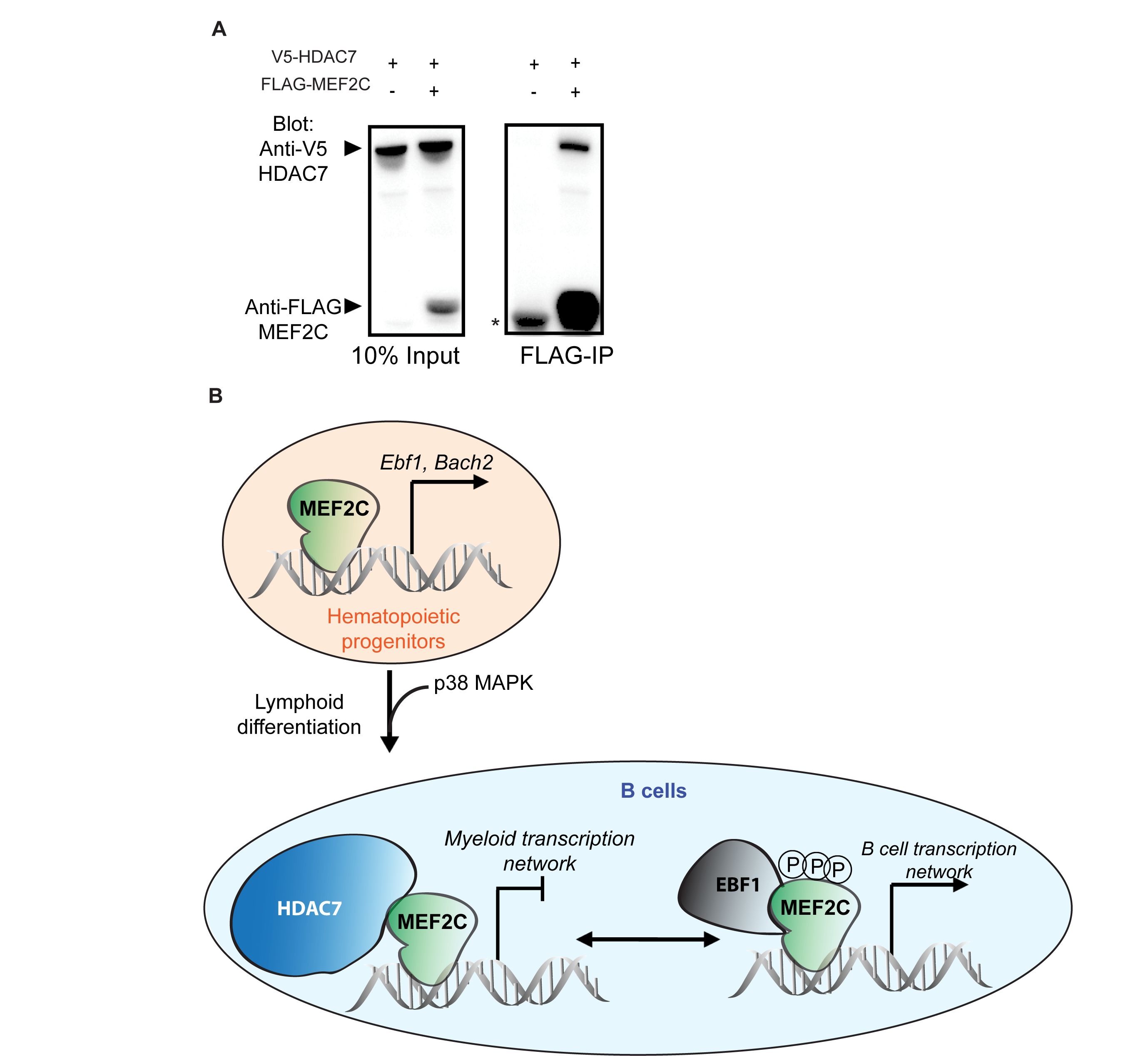

Supplement: S8 Fig — (A) FLAG-tagged WT MEF2C was co-transfected into 293T cells with V5-tagged HDAC7; FLAG-IP was blotted with anti-V5 antibody (top portion) or anti-FLAG antibody (bottom portion). Image was cropped from the same blot for clarity. Asterisk denotes heavy chain contamination, which is slightly smaller than MEF2C. (B) Model of B cell-specific transcription and lineage determination that requires MEF2C. (TIF) [file pgen.1005845.s008.tif]
