# Supplementary material for: MEF2C and EBF1 Co-regulate B Cell-Specific Transcription
Source: PLoS Genet. 2016 Feb 22;12(2):e1005845. doi: 10.1371/journal.pgen.1005845 (PMC4762780; doi:10.1371/journal.pgen.1005845)
Supplement: S1 Table — Results from two different ChIP experiments are shown here. The gene name, start, and end of each gene are bolded. The chromosome, start, end, and the score of each MACS-called peak are listed under each gene. All genes shown have binding overlap between EBF1 and both MEF2C datasets, except for the gene in parenthesis, which had binding overlap between EBF1 and only one of the MEF2C datasets. (PDF) [file pgen.1005845.s009.pdf]

MEF2C Rabbit Pre-B			
**Gene name Chromosome**	**Start**	**End**	**Score**
Mef2c	**83504036**	**83667079**	
Chr13	83439664	83439831	190.85
Chr13	83621470	83621623	104.81
Chr13	83662477	83662633	208.11
Ebf1	**44618134**	**45005172**	
Chr11	44592540	44592649	112.85
Chr11	44612716	44612794	81.12
Chr11	44622808	44622951	83.94
Chr11	44762985	44763049	106.29
Chr11	44813967	44814175	211.36
Chr11	44889124	44889241	152.63
Chr11	44910549	44910769	108.64
Chr11	44940837	44940969	68.54
Chr11	45005699	45005820	97.15
Chr11	45024128	45024225	136.53
Chr11	45071954	45072075	106.3
Foxo1	**52268337**	**52350109**	
Chr3	52216519	52216666	89.95
Chr3	52315577	52315766	246.24
Chr3	52350823	52351017	94.56
Ets1	**32696042**	**32757820**	
Chr9	32648797	32648973	223.71
Chr9	32744440	32744583	82.75
Chr9	32870615	32870750	96.83
Myb	**21124930**	**21160984**	
Chr10	21146678	21146832	82.14
Chr10	21165900	21166156	309.57
Bcl6	**23965052**	**23988612**	
Chr16	24067153	24067337	134.01
Pax5	**44531506**	**44710440**	
Chr4	44528216	44528345	83.48
Chr4	44583444	44583568	144.85
Chr4	44637829	44638030	117
Pou2af1	**51213690**	**51240079**	
Chr9	51227028	51227154	59.46
Chr9	51247409	51247706	336.88
Cobll1	**65088339**	**65238626**	
Chr2	65191595	65191681	68.54
Chr2	65213879	65214004	106.83
(Rag1)	**101638252**	**101649532**	
Chr2	101287448	101287569	70.9

Mbd2	**70568292**	**70626131**	
Chr18	70583585	70583755	91.77
Chr18	70630318	70630491	72.4
Notch2	**98013538**	**98150367**	
Chr3	98045968	98046043	91.24
Chr3	98088984	98089151	123.47
Lrrn3	**41451668**	**41486057**	
Chr12	41439250	41439374	77.98
Chr12	41499273	41499344	94.59

**MEF2C Goat Pre-B**			
**Gene name Chromosome**	**Start**	**End**	**Score**
Mef2c	**83504036**	**83667079**	
Chr13	83439552	83439755	95.14
Chr13	83632975	83633455	61.69
Chr13	83653838	83654020	164.43
Chr13	83662322	83662634	2070.39
Ebf1	**44618134**	**45005172**	
Chr11	44612617	44612850	56.85
Chr11	44621897	44622108	112
Chr11	44658207	44658405	133.46
Chr11	44696721	44696966	68.67
Chr11	44715900	44716133	57.05
Chr11	44814026	44814273	392.34
Chr11	44996874	44997171	81.08
Chr11	45009591	45009777	122.4
Chr11	45064699	45064925	80.81
Foxo1	**52268337**	**52350109**	
Chr3	52216428	52216610	127.07
Chr3	52306524	52306966	54.6
Chr3	52315642	52315867	112
Chr3	52378800	52379017	128.18
Ets1	**32696042**	**32757820**	
chr9	32648815	32649050	223.47
chr9	32656623	32657085	220.88
chr9	32668703	32669056	62.7
chr9	32787807	32788054	188.52
Myb	**21124930**	**21160984**	
Chr10	21110433	21110606	133.46
Chr10	21165985	21166229	161.72
Bcl6	**23965052**	**23988612**	
Chr16	24067184	24067383	171.48
Pax5	**44531506**	**44710440**	
Chr4	44528120	44528313	56.85

Chr4	44537805	44537999	85.07
Chr4	44579352	44579601	128.18
Chr4	44583373	44583559	112
Chr4	44642874	44643285	58.9
Chr4	44695136	44695444	91.02
Pou2af1	**51213690**	**51240079**	
Chr9	51247315	51247549	638.58
Cobll1	**65088339**	**65238626**	
Chr2	65182170	65182350	296.06
Chr2	65182660	65182837	138.4
Chr2	65191456	65191650	96.29
Chr2	65213825	65214002	144.76
(Rag1)	**101638252**	**101649532**	
Chr2	101519279	101519538	138.22
Chr2	101670033	101670225	105.34
Mbd2	**70568292**	**70626131**	
Chr18	70585261	70585490	84.24
Chr18	70594704	70595246	103.81
Chr18	70647441	70647789	59.65
Notch2	**98013538**	**98150367**	
Chr3	98056082	98056273	105.34
Chr3	98088939	98089204	149.61
Lrrn3	**41451668**	**41486057**	
Chr12	41499051	41499392	92.41

**EBF1 Goat Pre-B**			
**Gene name Chromosome**	**Start**	**End**	**Score**
Mef2c	**83504036**	**83667079**	
Chr13	83439712	83439862	98.6
Chr13	83440925	83440988	137.43
Chr13	83537014	83537107	83.27
Chr13	83629192	83629280	56.7
Chr13	83653840	83653912	100.37
Chr13	83662443	83662629	164.14
Ebf1	**44618134**	**45005172**	
Chr11	44612581	44612871	125.88
Chr11	44632112	44632199	83.27
Chr11	44691384	44691503	63.54
Chr11	44693875	44693975	69.48
Chr11	44772352	44772414	137.43
Chr11	44814015	44814186	347.42
Chr11	45012936	45013090	50.81
Chr11	45088511	45088577	321.26

Foxo1	**52268337**	**52350109**	
Chr3	52200141	52200266	66.43
Chr3	52286239	52286321	100.77
Chr3	52350842	52351018	119.33
Chr3	52358650	52358800	62.73
Chr3	52378822	52378914	83.27
Ets1	**32696042**	**32757820**	
Chr9	32688888	32688957	77.11
Chr9	32691959	32692051	56.7
Myb	**21124930**	**21160984**	
Chr10	21147185	21147313	79.38
Chr10	21149237	21149370	95.08
Chr10	21166025	21166147	215.94
Bcl6	**23965052**	**23988612**	
Chr16	24067176	24067326	161.27
Pax5	**44531506**	**44710440**	
Chr4	44528224	44528396	103.24
Chr4	44537510	44537715	83.57
Chr4	44549532	44549665	62.4
Pou2af1	**51213690**	**51240079**	
Chr9	51247399	51247608	1205.89
Cobll1	**65088339**	**65238626**	
Chr2	65219694	65219785	100.77
(Rag1)	**101638252**	**101649532**	
Chr2	101616586	101616678	137.43
Chr2	101623441	101623547	63.54
Chr2	101670178	101670286	54.99
Mbd2	**70568292**	**70626131**	
Chr18	70573544	70573681	81.13
Chr18	70593291	70593374	100.77
Chr18	70615149	70615269	66.43
Chr18	70630347	70630509	93.26
Chr18	70630878	70631016	64.5
Notch2	**98013538**	**98150367**	
Chr3	98056048	98056165	54.99
Chr3	98066265	98066344	100.77
Chr3	98088922	98089122	287.85
Lrrn3	**41451668**	**41486057**	
Chr12	41454096	41454200	156.47
Chr12	41491143	41491206	132.18
Chr12	41499288	41499434	207.7
