# Supplementary material for: MEF2C and EBF1 Co-regulate B Cell-Specific Transcription
Source: PLoS Genet. 2016 Feb 22;12(2):e1005845. doi: 10.1371/journal.pgen.1005845 (PMC4762780; doi:10.1371/journal.pgen.1005845)
Supplement: S2 Table — Results from two different ChIP-experiments are shown here. The gene name, start, and end of each gene are bolded. The chromosome, start, end, and the score of each MACS-called peak are listed under each gene. (PDF) [file pgen.1005845.s010.pdf]

MEF2C Rabbit HPC			
Gene Name Chromosome	Start	End	Score
Mef2c	**83504036**	**83667079**	
Chr13	83095796	83095948	189.05
Chr13	83439637	83439765	115.57
Chr13	83592757	83592931	120.22
Chr13	83633042	83633259	84.88
Chr13	83662358	83662635	863.11
Ebf1	**44618134**	**45005172**	
Chr11	44811824	44811926	151.5
Chr11	44889116	44889226	94.98
Foxo1	**52268337**	**52350109**	
Chr3	52216499	52216678	98.12
Chr3	52350832	52351037	102.65
Ets1	**32696042**	**32757820**	
Chr9	32648874	32648995	179.77
Myb	**21124930**	**21160984**	
Chr10	21165944	21166178	133.8
Pou2af1	**51213690**	**51240079**	
Chr9	51247396	51247565	198.57
Bcl11a			
Chr11	24207463	24207633	107.14
Bach2	**32417435**	**32586108**	
Chr4	32202478	32202651	102.72
Notch2	**98013538**	**98150367**	
Chr3	98056079	98056188	94.48
Chr3	98088953	98089124	76.71

MEF2C Goat HPC			
Gene Name Chromosome	Start	End	Score
Mef2c			
Chr13	83095823	83096020	3100
Chr13	83140723	83140818	2610.74
Chr13	83187537	83187664	3100
Chr13	83206750	83206825	2269.81
Chr13	83210243	83210431	3100
Chr13	83238826	83238951	1468.79
Chr13	83281792	83281958	3100
Chr13	83439660	83439838	3100
Chr13	83491920	83492060	1760.66
Chr13	83512647	83512731	2991.2
Chr13	83524098	83524213	1193.92

Chr13	83585718	83585788	2009.09
Chr13	83621455	83621606	3100
Chr13	83662524	83662636	3100
Chr13	83667139	83667188	3100
Ebf1	**44618134**	**45005172**	
Chr11	44592532	44592592	3047.91
Chr11	44605936	44606050	1532.59
Chr11	44612662	44612913	3100
Chr11	44622794	44622858	2501.55
Chr11	44622903	44622954	3100
Chr11	44640403	44640456	3228.29
Chr11	44653594	44653686	1493.4
Chr11	44658206	44658281	3100
Chr11	44715967	44716052	1532.64
Chr11	44762872	44763041	2817.81
Chr11	44773083	44773225	3100
Chr11	44811770	44811908	3034.26
Chr11	44813998	44814154	3100
Chr11	44823311	44823390	1777.19
Chr11	44844046	44844110	2644.66
Chr11	44847634	44847753	1366.37
Chr11	44858405	44858466	2780.85
Chr11	44861720	44861772	3178.93
Chr11	44889158	44889281	1126.1
Chr11	44897430	44897479	3100
Chr11	44910591	44910740	3100
Chr11	44931970	44932109	1516.3
Chr11	44936642	44936741	1568.26
Chr11	44940835	44940936	3100
Chr11	44983627	44983701	2259.12
Chr11	44993469	44993554	1567.18
Chr11	45005785	45005854	2004.4
Chr11	45023931	45024050	1441.57
Chr11	45024153	45024264	1468.79
Chr11	45046365	45046476	2250.59
Chr11	45062756	45062816	3100
Chr11	45071947	45072030	3100
Foxo1	**52268337**	**52350109**	
Chr3	52216535	52216714	3100
Chr3	52271424	52271506	3100
Chr3	52287927	52288029	1577.57
Chr3	52315620	52315764	3100
Chr3	52350828	52350998	3100
Chr3	52364798	52364865	2517.61
Chr3	52378827	52378917	3100

Ets1	**32696042**	**32757820**	
Chr9	32637873	32637935	2684.07
Chr9	32648810	32648968	3100
Chr9	32656157	32656282	1468.79
Chr9	32656944	32657053	3100
Chr9	32673412	32673553	1264.91
Chr9	32700555	32700605	3100
Chr9	32706803	32706897	2471.63
Chr9	32722818	32722908	1560.08
Chr9	32751736	32751836	2189.53
Chr9	32787956	32788009	3208.53
Myb	**21124930**	**21160984**	
Chr10	21150192	21150287	1957.68
Chr10	21165912	21166138	3100
Bcl6	**23965052**	**23988612**	
Chr16	24056442	24056611	3100
Chr16	24065233	24065309	3100
Chr16	24067218	24067310	3100
Il7r	**9506159**	**9529876**	
Chr15	9491386	9491435	3100
Pax5	**44531506**	**44710440**	
Chr4	44528216	44528401	3100
Chr4	44537490	44537620	3100
Chr4	44537706	44537772	2665.78
Chr4	44559647	44559758	1468.79
Chr4	44579479	44579565	3100
Chr4	44583411	44583578	3100
Chr4	44590471	44590537	3100
Pou2af1	**51213690**	**51240079**	
Chr9	51217413	51217571	3100
Chr9	51231893	51231957	2534.67
Chr9	51247415	51247608	3100
Bcl11a	**24078056**	**24168435**	
Chr11	24106460	24106513	3100
Chr11	24114279	24114386	1492.4
Cobll1	**65088339**	**65238626**	
Chr2	65182278	65182403	3100
Chr2	65189547	65189602	3100
Chr2	65191140	65191219	3100
Chr2	65191559	65191692	3100
Chr2	65213872	65213946	2291.31
Bach2	**32417435**	**32586108**	
Chr4	32202450	32202620	3100
Chr4	32202695	32202762	2627.51
Chr4	32222517	32222699	3100

Chr4	32233919	32234037	1420.35
Chr4	32258443	32258508	3100
Chr4	32293886	32293935	3203.53
Chr4	32304956	32305023	2175.02
Chr4	32365768	32365866	3100
Chr4	32389660	32389919	3100
Chr4	32463105	32463180	1741.96
Chr4	32488259	32488390	1233.84
Lig4	**9970020**	**9976323**	
Chr8	9898924	9899063	3100
Chr8	9930221	9930353	1405.1
Chr8	9988032	9988169	2590.44
Rag1	**101638252**	**101649532**	
Chr2	101613727	101613792	3100
Chr2	101628511	101628560	3100
Chr2	101641928	101642010	1927.44
Chr2	101644469	101644660	3100
Chr2	101649027	101649077	3100
Chr2	101670157	101670219	2432.37
Mbd2	**70568292**	**70626131**	
Chr18	70572856	70572990	1153.83
Chr18	70576866	70576948	2916.09
Chr18	70578672	70578751	3100
Chr18	70630405	70630506	1361.46
Notch2	**98013538**	**98150367**	
Chr3	98039681	98039799	1193.92
Chr3	98056093	98056171	3100
Chr3	98084272	98084397	1193.92
Chr3	98088972	98089100	3100
Lrrn3	**41451668**	**41486057**	
Chr12	41439226	41439374	3100
Chr12	41439527	41439616	1582.03
Chr12	41441221	41441310	1540.18
Chr12	41453020	41453100	1729.38
Chr12	41454084	41454230	3100
Chr12	41466859	41466911	3100
Chr12	41499285	41499421	2474.89
