# Supplementary material for: MEF2C and EBF1 Co-regulate B Cell-Specific Transcription
Source: PLoS Genet. 2016 Feb 22;12(2):e1005845. doi: 10.1371/journal.pgen.1005845 (PMC4762780; doi:10.1371/journal.pgen.1005845)
Supplement: S3 Table — Genomic sequences of murine Il7ra, Ebf1, and Foxo1 genes that were cloned into pGL4.23 luciferase reporters are listed here. Bolded are MEF2C consensus binding sites. Underlined are potential EBF1 binding sites or half sites. Bolded and underlined are sequences that were mutated. (PDF) [file pgen.1005845.s011.pdf]

Il7ra

GGGGATTGCATTGAATCTGTAGATTGCTTTTGGCAAGATAGCCATTTTTACA
ATGTTGATCCTGCCAATCCATGAGCATGGGAGATCTTTCCATCTTCTGAGAT
CTTCTTTAATTTCTTCCTTCAGAGATTTGAAGTTTTTATCATACACATCTTTCA
CTTCCTTAGTTAGAGTCACGCCAAGATATTTTATATTATTTGTGACTATTGAG
AAGGGTGTTGTTTCCCTAATTTCTTTCTCAGCCTGT**TTATTCTTTGTATAGAG**
AAAGGCCATTGACTTGTTTGAGTTTATTTTATATCCAGCTACTTCACCGAAG
CTGTTTATCAGGTTTAGGAGTTCTC**TGGTAGAATTTTTAGGGTCACT**TATAT
ATACTATCATATCATCTGCAAAAAGTGATATTTTGACTTCCTCTTTTCCAATTT
GTATCCCCTTGATCTCCTTTTGTTGTCGAATTGCTCTGGCTAATACTTCAAGT
ACTATGTTGAAAAGGTAGGGAGAAAGTGGGCAGCCTTGTCTAGTCCCTGAT
TTTAGTGGGATTGCTTCCAGCTTCTCTCCATTTACTTTGATGTTGGCTACTGG
TTTGCTGTAGATTGCTTTTATCATGTTTAGGTACGGGCCTTGAATTCCTGATC
TTTCCAAAACTTTTATCATGAATGGGTGTTGGATCTTG

Ebf1

GGCTGAACTAAGCTTTGGTGAATTCAGATACCTTGACT**TGGGGACCTGGAA**
ATAGCAGTGTTTTTCATGGGGACAGTGAGGTCCTCTTTAAAAGAGAAGGCA
AGACAGAATCTAAAGATACGCTACTGGATTTTTAGAGGTACTGTGGAATGTC
TCAAAGATTCTCAAATGCAGGAATTCTGGGGTTCTAGGCTGTCCTCTTGACA
GATGACATGTCTCTGAGCCATTGGGATTGACTGAGTCACATCCTAGCTCTAC
TGATGACTGTGTGTAGCCTCTGCCTTTTAATGTTACTGTGTAAAGACTGGGA
AGACCTGTTAGATCTGGGATGTGGATTAAATTAACTGAAGTTTATGATGGAT
GTTCTTGGTGTCATAACTAGTTCCTATTAAGCGACAGTTGCTATTGTGCTCAA
TGCTTTCATGGCTGCTGTTATCATCATCAAGACTGAGAGACTTGCTTTAGGG
ATATTGCCTGTTGAGACTTTCTCTAGGGAGTACAAGTCAATATACCAGTTGA
GAAACAGGTGATGACTACCTGGTAGAAAAATAACCAAGGCCTGCATACATG
CTACTTATCTCCAGACTCTGGGCCCAGGAATTCACACAAAAAAGAACAATAT
GGGCAGCTTTTCTCAATGCCCCATGGCAGTTCGTATTATCTTCTCTCTTTTAA
ACTGCATTTAGTATTGATCAAAATTTGATCTCCCTAGGAAAGAAAAATGTATT
GGAGGGTGATAAA**GGGACAAATAGCA**ATATGATTTGTTTAGAA**CCC**CCAGC
TCCCAGCATCTTCCAGATCACCTCAATCACCCAGAGGT

Foxo1

TAGTAGCCTGGGCTGCAGTTTGTGTTCTCTTAGTGTCTGTATAACATCTGTC
CAGGCTCTTCTGGCTTTCATAGTCTCTGGTGAAAAATCTGGTGTAATTCTGA
TAGGCTTGCCTTTATATGTTACTTGACCTTTTTC**CCTTACTGCTTTTAGTATT**
CTATCTTTATTTAGTGCATTTGATGTTCTGATTATTATGTGTCGGGAGGAATT
TCTTTTCTGGTCCAGTCTATTTGGAGTTCTGTAGGCTTCTTGTATGTTCATAT
GCATCTCATTCTTTAGATTTGGGAAGTTTTCTTCAATAATTTTGTTGAAGATG
TTTGCTGGACCTTTGAGTTGAAAATCTTCATTCTCATCCACTCCTATTATCCG
TACGTTTGGTCTTCTTATTGTGTCCTGGATTTCCTGGATATTTTGAGTTAGGA
TCTTTTTGCATTTTCCATTTTCTTTGATTGTTGTGCCGATGTTCTCTATGGAAT
CTTCTGCACCTGAGATTCTCTCTTCCATCTCTTGTATTCTGTTGCTGATGCTC
AAATCTATGGTTCCAGATTTCTTTCCTAGGGTTTCTATCTCTAGTGTTGCCTC
GCTTTGAGTTTTCTTTATTGTGTC**TACTTCCCTTTTTAGGTCTAGT**ATGGTTT

TGTTCATTTCCATCACCTGTTTGTATGTTTTTTCCTCTTTTTCTGTAAGGACTT
CTACCTGTTTGATTGTGTTTTCCTGTTTTTCTTTAAGGACTTGTAACTCTTTAG
CAGTGTTCTCCTGTATTTCTTTAAGTGATTTATTAAAGTCCTTCTTGATGTCCT
CTACCATCATCATGAGATATGCTTTTAAATCTAGGTCTAGGTTCTCAGGTGT
GTTGGGGTCCCCTGGACTGGGCGAAGTGGGTGTGCTGG**G**TTCTGGTGATG
GTGAGTGGTCTTGGTTCCTGTTAGTAAGATTCCTCCGTTTACCTTTCGCCAT
CTGGTAATCTCTGGAGTTAGTAGTTATAGTTGACTCTGTTTAGAGATTGTTCT
TCTGGTGATTCTGTTACCGTCTATCAGCAGACCTGGGAGACAGATTCTCTCC
TCTGAGTTTCAGTGCTCAGAGCACTCTCTGCTGGCAAGCTCTCTTACAGGG
AAGGTGCGCAGATATCTTGTATTTGGACCTCCTCCTGGCCGAAGAAGAAGG
CCCAAAACAGGACCTTTCTCAGACACTGTGTTGCTTTGGCAGTTCCCAGGT
GGTACAGACTCTCACCTAAGCAGACTAAATTCCTAAGTTCCTTGGAGTCCCG
GGACCAAGATGGCGACCGCTGCTGCTGTGGCTTAGGCCGCCTCCCCAGCC
GGGTGGGCACCTGTCCTCCGGTCCGGAAGGTGGCCGGCTGTCCCCGGCC
CACACAGGGTGCTGCCTCAGCCCCTCTGTGCTTCTGCCTGTTCCAGAAGCT
GTCAGGTTCTCTGGCGCACCCTCTCACCTGTTCAGACTAATTTCCTAAGTTC
GGCGGGTCCCGGACCAAGATGGCGACCGCTGCTGCTGTGGCTTAGGCCGC
CTCCCCAGCCGGGCGGGCACCTGTCCTCCGGTTCGGACGGTGGCCGGCT
GTCCCCGGCCCACACAGGGTGCTGCCTCAGCGCCTCTGTGCTTCTGCCTG
TTCCAGAAGCTGTCAGGTTCTCTGGCGCACCCTCTCACCTGTTCAGACTAAT
TTCCTAAGTTAGGCGGGTCTCGGACCAAGATGGCGACCGCTGCTGCTGTG
GCTTAGGCCGCCTCCCCAGCTGGGTGGGCACCTGTCCTCCGGTCCGGAAG
GTGGCCGGCTGTCCCCGGCCCACACAGGGTGCTGCCTCAGCCCCTCTGTG
CTTCTGCCTGTTCCAGAGGCTGTCAGGTTCTCTGGCGCACCCTCTCACCTG
TTCAGACTAATTTCCTAAGTTCGGCGGGTCCCGGACCCTACAAAATTTTTAA
ATTTTGTTTTTCTTGAGGATGTCACACATGAGTATACTGTATAAATTGTTTTCA
TCTGTCCGTCTCTCTGACTCCTTCCATTTTCCTCTCCTGTCTCTCAAATTCAT
GACCTCCTCTATAGCTAGTGTTGTGCATACATGTGTGTGTACATACCACACA
CACACACACACACACACACACACACACACACACACACACACTACTGAGTCC
AGTGCTCATCTCTGGAGAAAACTGACTCTCATTCCTTAGCAGCCATTGCTTG
CCTGAAGTTCTTCATCGAAGGCTTGGGGCCTTGTTAAATTGGTGGCACACG
CCTTAATCTCAGCACTCGGGAGGCAGAGGCAGAAGCAGGTGGATTTCTGAG
TTTGAGGCCAGCCTGGTCTACAAAGTGAGTTCCAGGACTGCCAGGGCTATA
CAGAGAAACTCTGTATCGAAAAACAAAAACAAAAACAAAAAACAAAAAAAAA
AAAAATTTACCCATCCATGTTGGGATATTTCCTGGTGTGGTCTTGTATAGGT
CTAATGTGGGCAAGCCATTGTTGAGAGCTCATGGGTGCAGCATCTCTGTCA
TGTCTGGAAGACACTATCTCATAGCAGTTGTCTGGTCGTCTGGCCCCCAGA
GTCTTTCTGCCTCTTTCCTTTATGTTACTTGAGCATTGGGTATAGCAGTTGTG
TTATAAATGTCCTAATAGGGTCTGGGCTCCTCACAGTTACTTCCTCTCTGCC
TTTTTGACCAGTTATGGATCGCTATAAGTAGTTTCTACTGCAAAAAGACACTT
CATAGTGGAGGGTGAGAACAGTACTTACCCAGAGAGCATTGGCACAGCTGT
CATCTGGCCACACCCAGCATTTTCCCTTGGTTCTTGTTCTCAGGTCCTTATG
CTTGCAAGGCAAATACCTTACCAACCGAGTGTCCATATAATCTTTGTTTGTTT
GAGACAGGGTCTCTCTTTGTAGTCTTGACTGTTCTGGACTTCAGTATGTAGA
CCAGGTAGACCAAACCTTGAATTCACAGAGCTCCACCTGGCACTGCTTCTC
AAGTGCTGGGATTAAAGGCATTTGCCACTACGTTCAGCATAATATATACTCT

TAATAAAGATTTATTGTATAGGAGGCTAGGAATAGGTCTTCTTCCAGGTCCA
GTTATACCACTCTTGAACATATACTCGAAGGACTTTATATCCTACTACAGAG
GCATATGCTCATTTGTGTTTATTGCTGCTCTATAAACAATAACCAGAATTTGG
AAACAGCCTAGATGTCCATCAACAGATAAATGGATAATGAAAATGTGGTACA
TTTATACAATGGAATATTACTCTGCTGTTAAAAGAAATGAAATCATGAAACTC
TCAGGTAAATGGATGAAGATAGGAAAAAAAATTCATCCCGAGTGAGGTAATA
CAGACCCCAAAAGACAAACATGGTATGCATTCTCTTTTGTGTGGATGTTAGC
TTTTAAGCTTTCAATAGACATGCTGCAGAGGTTAGGCATAAAGGGCTAGAGG
ACTGGGAATGGGGAAATAGAATATATTTCTAGTTATGGAAAGATGTGGAGG
GGAGTCTAGAATGGAGAGGGGACATAAGGGGGTAAGGAGTGGGATACAGA
GATGGACAGCTAACACTAAGGGCCATTTGAGGGGTCATATGGAAATCTACT
ATAGTACAAGCTTCTTAAAATATATTCATATACAAAATAAATCTAAATGACATC
ACTAAACAACAGGGGAGACAAAGCCCAACCAACCAAGACATCTCCCAGTGA
AATCTCCAGTGCCAGAAATGGGTTATATAGCTTATTGAGTTGTTGGCCCAAT
GAACCCCATGGAACCCCCCAAACAACTCAGGATATTTCCAAGTCTATTGGTT
GCTCTTCACAAATTGATGCTAAGGCCTTATTGTTGAAGACAACACCTATGTA
TCTTATTGAACATGGGAAAGTCAAATTATTGCCTACCTAGAGCCTTCAGCCC
TATTGACTAGTATGGATGGTTCCGGAAGGTACTCTGCAACCCAGCCACAAC
CCTTTGATCGACAACAAAAACCTGCCTGCCTGACATGCTGGCGCAATGGTG
GCACGGAAGTCGCAAGAGTAACCAAATGTTTTCAGATTATATTTTAAGTGCG
TTCCATGAGGTGGAGCACTGTTCAGGTAGCTAAGAATCTGAGACTAGATAC
GCCAGGGACCTAGGGGAGAAGCTAATTCTGTTGTTCTACTACAGGAATATG
GCAATAAACTGACTCCTAATGGCCTTCTCCTATGTACATAGATCAGTTTCTTA
GTTAGCCATCACCAGAGAAGCTTCCTCCTGCAGTAGATGGGAACAAACACA
GAGACTCACCACTGGACAATATGCAGAAAATAAAGATATCAAACTCTCCACT
CACAGCTCACGAAATTATGTAGAAGAAGCAGAAAGACTCTAAGAACCAGTG
GGTTTCCCCCTCAATTCCCGGAATTCCTCTCAATTCCAGTTGTTTCTCCCAG
TAATTTCTCTTTTGCTCCCTCCCACCTGATCCCCGCCTGGTCTTCTGCAGAA
TCTATTCTATTTCCCCTGCCCTGAGAGGTCCTTTTGTCCCTGCCTTAAGCCC
TCTTTGTTACTTAGCTTCTCTAGGTCTGTATCATGACTGTCTT**TTATCCACTA**
ATATTAGCTAATATCCACTTATAAGTGAGCATATATCATATTTGTTTTTCTGG
GTCTCGGTTACC
