# Supplementary material for: MEF2C and EBF1 Co-regulate B Cell-Specific Transcription
Source: PLoS Genet. 2016 Feb 22;12(2):e1005845. doi: 10.1371/journal.pgen.1005845 (PMC4762780; doi:10.1371/journal.pgen.1005845)
Supplement: S4 Table — (PDF) [file pgen.1005845.s012.pdf]

**Gene**	**Forward**	**Reverse**
Mef2c-1	AAGAAACACGGGGACTATGG	ACAGCTTGTTGGTGCTGTTG
Mef2c-2	GTGCTGTGCGACTGTGAGAT	TTGAGGCCCTTCTTTCTCAA
Mef2c-3	CGAGGATAATGGATGAGCGTA	CATGTCAGTGCTGGCGTACT
Ebf1-1	CCCTCCAACTGCAGTAGCTC	TTTCACATGGGAGGGACAAT
Myb	TGTCCTCAAAGCCTTTACCG	CCGTCATCTGGTCCTCTGTC
Ets1	ATCCAGCTGTGGCAGTTTCT	CCACGGCTCAGTTTCTCATA
Flt3	GAACCCTTACCCTGGCATTC	TCAGGTTGGGGAAGGATG
Foxo1	TCCTGGGCCAAAATGTAATG	GGTTCATGGCAGATGTGTGA
Cxcl9	TTTTCCTCTTGGGCATCATC	TGTTGCAATTGGGGCTTG
Csf2ra	GACACGAGGATGAAGCACTG	GAACCTCCTGCACGTCACTC
Csf3r	GTAGCCTGAGCTCCTGGTTG	GGCTACCATTCCCAGAGCTT
Csf1r	CTGGGAGATCTTCTCGCTTG	TCTGTTGGAAGGTGGGTCTT
Rag1	TTTCACAAAACCTTGGCACA	CAGCCAGTGATGTTTCAGGA
Il7ra	GCCTAGTCTCCCCGATCATA	TCTCCAACTCCTCTGGCTGT
Sfpi1	GAGAAGCTGATGGCTTGGAG	GCTTGGACGAGAACTGGAAG
C/ebpa	GGGACCATTAGCCTTGTGTG	AGCATAGACGTGCACACTGC
Gapdh	TGTGCAGTGCCAGCCTCGTC	TGAAGGGGTCGTTGATGGCAACA
RNP	TTGCAGGAAGAAGAGAGAGAGAGG	TGCCAAAGTCCAGGAGCTTCAG
Bcl11b	GACTCAGGGTGAGGGTCAGA	AAGCCATGTGTGTTCTGTGC
Pou2af1	CCAGCCTGGCTTTGAACTTA	CGAGTCTGTAGTGCCTGCTG
Jun	TATTTTGGGGAGCATTTGGA	CTCTGGGTCAGGAAAGTTGC
